# Factors Influencing Recanalization After Mechanical Thrombectomy With First-Pass Effect for Acute Ischemic Stroke: A Systematic Review and Meta-Analysis

**DOI:** 10.3389/fneur.2021.628523

**Published:** 2021-04-09

**Authors:** Xuesong Bai, Xiao Zhang, Jie Wang, Yinhang Zhang, Adam A. Dmytriw, Tao Wang, Ran Xu, Yan Ma, Long Li, Yao Feng, Carolina Severiche Mena, Kun Yang, Xue Wang, Haiqing Song, Qingfeng Ma, Liqun Jiao

**Affiliations:** ^1^China International Neuroscience Institute, Beijing, China; ^2^Department of Neurosurgery, Xuanwu Hospital, Capital Medical University, Beijing, China; ^3^Neuroradiology & Neurointervention Service, Brigham and Women's Hospital, Harvard Medical School, Boston, MA, United States; ^4^Pontifical Bolivarian University, Medellín, Colombia; ^5^Department of Evidence-Based Medicine, Xuanwu Hospital, Capital Medical University, Beijing, China; ^6^Medical Library, Xuanwu Hospital, Capital Medical University, Beijing, China; ^7^Department of Neurology, Xuanwu Hospital, Capital Medical University, Beijing, China; ^8^Department of Interventional Neuroradiology, Xuanwu Hospital, Capital Medical University, Beijing, China

**Keywords:** acute ischemic stroke, mechanical thrombectomy, first pass effect, influencing factors, systematic review, meta-analysis

## Abstract

**Background:** First-pass effect (FPE) is increasingly recognized as a predictor of good outcome in large vessel occlusion (LVO). This systematic review and meta-analysis aimed to elucidate the factors influencing recanalization after mechanical thrombectomy (MT) with FPE in treating acute ischemic stroke (AIS).

**Methods:** Main databases were searched for relevant randomized controlled trials (RCTs) and observational studies reporting influencing factors of MT with FPE in AIS. Recanalization was assessed by the modified thrombolysis in cerebral ischemia (mTICI) score. Both successful (mTICI 2b-3) and complete recanalization (mTICI 2c-3) were observed. Risk of bias was assessed through different scales according to study design. The *I*^2^ statistic was used to evaluate the heterogeneity, while subgroup analysis, meta-regression, and sensitivity analysis were performed to investigate the source of heterogeneity. Visual measurement of funnel plots was used to evaluate publication bias.

**Results:** A total of 17 studies and 6,186 patients were included. Among them, 2,068 patients achieved recanalization with FPE. The results of meta-analyses showed that age [mean deviation (MD):1.21,95% confidence interval (CI): 0.26–2.16; *p* = 0.012], female gender [odds ratio (OR):1.12,95% CI: 1.00–1.26; *p* = 0.046], diabetes mellitus (DM) (OR:1.17,95% CI: 1.01–1.35; *p* = 0.032), occlusion of internal carotid artery (ICA) (OR:0.71,95% CI: 0.52–0.97; *p* = 0.033), occlusion of M2 segment of middle cerebral artery (OR:1.36,95% CI: 1.05–1.77; *p* = 0.019), duration of intervention (MD: −27.85, 95% CI: −42.11–13.58; *p* < 0.001), time of onset to recanalization (MD: −34.63, 95% CI: −58.45–10.81; *p* = 0.004), general anesthesia (OR: 0.63,95% CI: 0.52–0.77; *p* < 0.001), and use of balloon guide catheter (BGC) (OR:1.60,95% CI: 1.17–2.18; *p* = 0.003) were significantly associated with successful recanalization with FPE. At the same time, age, female gender, duration of intervention, general anesthesia, use of BGC, and occlusion of ICA were associated with complete reperfusion with FPE, but M2 occlusion and DM were not.

**Conclusion:** Age, gender, occlusion site, anesthesia type, and use of BGC were influencing factors for both successful and complete recanalization after first-pass thrombectomy. Further studies with more comprehensive observations indexes are need in the future.

## Introduction

Stroke is the second-leading cause of global morbidity and mortality (1, 2). Mechanical thrombectomy (MT) has been widely used to treat acute ischemic stroke (AIS) patients and has proved superior over intravenous tissue-type plasminogen activator (tPA) by several landmark randomized trials (RCTs) (3–6). Thus, the American Heart and American Stroke Association recommends MT as the first-line therapy for selected AIS patients with proximal artery large vessel occlusions (LVO) (2).

However, some trials showed that functional independence in AIS patients is only around 50% even with a high recanalization rate of over 70% (3, 6). Thrombectomy with first pass effect (FPE), an emerging new metric, is strongly correlated with improved functional outcomes (7–10). Thrombectomy with FPE may have many advantages such as less vessel wall injury, lower risk of clot fragments, and decreased time to reperfusion (8, 11). Also, FPE is associated with better outcomes than MPE after achieving successful or complete recanalization (12). Thus, identifying factors influencing FPE could help clinicians and interventionalists maximize the benefit of MT through suitable patient selection and pre-interventional risk modification. There are many studies seeking to explore this phenomenon, but with inconsistent results (7, 8, 11, 13–20). For example, balloon guide catheters (BGC) and non-internal carotid artery (ICA) terminus occlusion were correlated with FPE in the study of Zaidat et al. (7), but factors such as older age, a lower systolic blood pressure, and conscious sedation were not (17).

Thus, this systematic review and meta-analysis seeks to summarize the current literature investigating influencing factors of thrombectomy with first pass and elucidate associations with it.

## Methods

This study was reported in conformity to the criterion of Preferred Reporting Items for Systematic Reviews and Meta-Analyses (PRISMA) (21).

### Search Strategy

Eligible studies were independently searched by two reviewers from the following databases: MEDLINE, EMBASE, Web of Science, and the Cochrane Library. Clinical trial registers were also searched as potential sources. The included studies were restricted to the publication time before October 31, 2020, and the English language. The following key words were used: “acute ischemic stroke,” “mechanical thrombectomy,” “endovascular thrombectomy,” “first pass effect,” “first attempt,” “recanalization.” A search strategy Table is presented in detail in the online supplementary material (online Supplementary Table 1).

### Study Selection

#### Patient Selection Criteria

Inclusion criteria included age ≥18 years with AIS due to large vessel occlusion, including the anterior or posterior circulation. Arterial occlusion was confirmed by computed tomographic angiography (CTA), magnetic resonance angiography (MRA), or digital subtraction angiography (DSA). Exclusion criteria included patients with baseline pre-stroke mRS score ≥3 and artery occlusion of non-atherosclerotic etiology such as dissection, moyamoya disease, vasospasm, or vasculitis. Patients with ICH, significant cerebellar mass effect, and acute hydrocephalus on CT or MRI before the onset of stroke were also excluded.

#### Definitions

FPE was defined as achieving successful or complete recanalization by MT after first pass regardless of thrombectomy device, such as contact aspiration and stent retriever. By contrast, non-FPE was defined as failure to achieve successful or complete recanalization by MT after first pass using different thrombectomy devices, such as contact aspiration and stent retriever.

#### Outcome

The primary outcome was successful recanalization with FPE, and secondary outcome was complete recanalization with FPE. The definitions of successful recanalization and complete recanalization were up to modified thrombolysis in cerebral ischemia (mTICI) score of 2b-3 and 2c-3 respectively after MT by post-interventional DSA as per usual convention (22, 23).

#### Studies

RCTs and observational studies including cohort studies, case-controlled studies, and case series where the number of patients exceeded 10 were included to avoid type II errors from low power (24, 25). Case reports, conference abstracts, or case series reports with the number of included patients <10 were excluded.

### Selection of Studies and Data Extraction

Studies which qualified were extracted by two independent reviewers (YZ and RX). In the initial stage of screening, titles, keywords, and abstracts were screened, and irrelevant studies were then excluded. Subsequently, reviewers obtained the full articles of all the remaining studies and checked the full texts to ascertain the included variables. In addition, the reasons for inclusion or exclusion of studies after full-text check were recorded. Disagreement in study selection between two reviewers was resolved by a third reviewer (TW).

Two reviewers independently (LL and XW) extracted the data according to a standardized data extraction form. The extracted information of included studies was as follows: (1) Authors, publication time, country, number of patients in FPE and non-FPE groups, inclusion and exclusion criteria; (2) Mean age, gender, medical history, site of occlusion by angiography, admission NIHSS score, baseline ASPECTS, MT strategy, use of tPA, and procedural times. The resolution of disagreement regarding data extraction was achieved through assistance of a third reviewer (TW). For missing or unclear data in included studies, effort was made to contact the corresponding authors by e-mail in order to best guarantee the accuracy of data.

### Assessment Risk Bias and Heterogeneity

Two reviewers (YF and CSM) independently assessed the risk of bias of each included study. The Cochrane Collaboration criteria were applied for RCTs, and the Newcastle–Ottawa scale was used for observational studies, including cohort studies and case–control studies (26). For case series, the method described in Methodological Quality and Synthesis of Case Series and Case Reports was applied (27). The heterogeneity of pooled outcomes was evaluated by the *I*^2^ statistic. The *I*^2^ statistic that was >60% demonstrated high heterogeneity, and the DerSimonian and Laird method for random-effect estimation was performed for pooling outcomes. If heterogeneity was mild or moderate, the Mantel–Haenszel method for fixed-effect estimation was applied. In instances where heterogeneity of outcomes and sufficient studies were high, we conducted subgroup analysis by site of occlusion, such as anterior circulation or posterior circulation. The meta-regression and sensitivity analysis were also used to explore the potential sources of heterogeneity.

### Statistical Analysis

The STATA statistical software package (version 15.0, Stata Corp, College station, Texas, USA) was used for all data analysis and heterogeneity assessments. For dichotomous data, we adopted odds ratios (OR) with 95% confidential interval (CI), and the mean difference (MD) with 95% CI was used for continuous data. The standard of *p*-value <0.05 was regarded as statistically significant. If the number of included studies was more than 10, publication bias was assessed by visualization of a funnel plot.

## Results

### Study Selection and Study Characteristics

**Figure 1:**
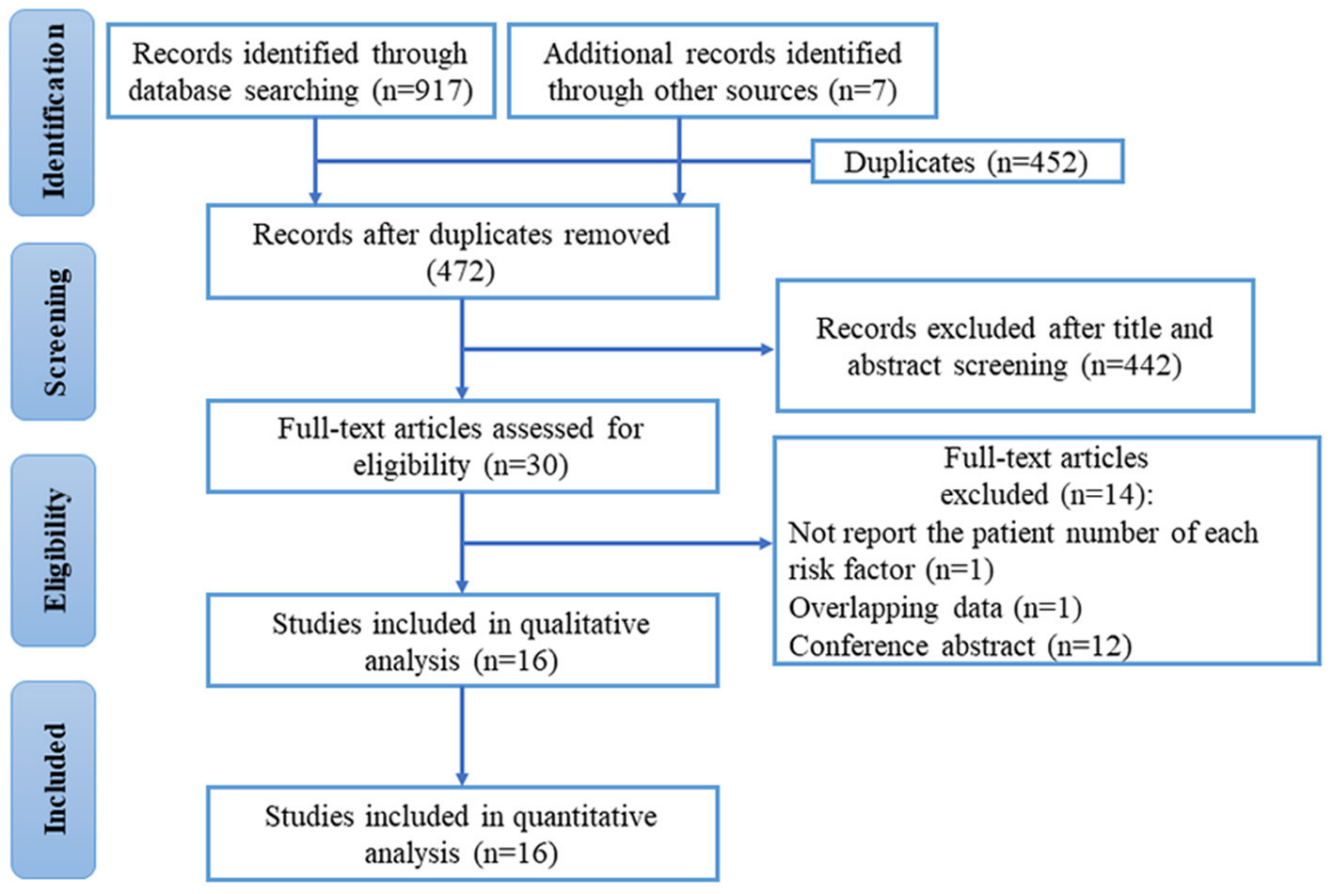
Flow diagram for systematic review and meta-analysis.

There were 924 records identified through the main database and clinical trials registers, and 16 studies were finally eligible for inclusion in the qualitative and quantitative analysis. The flow diagram of study selection is demonstrated in Figure 1.

Table 1 depicts the characteristics of included studies. A total of 16 studies and 6,095 patients were eligible according to inclusion criteria. Among them, 2015 (33.1%) patients achieved recanalization with FPE. All studies were published after 2016, seven conducted in Europe, five conducted in North America, and four in Asia. There were seven multicenter studies, and the remaining were single-center investigations. The number of patients in each study ranged from 50 to 1,832, and the numbers of male and female patients were essentially equal [2,929 (50.05%) vs. 2,923 (49.95%)]. Mean NIHSS scores ranged from 2 to 28. The location of occlusion by angiography was mostly within the anterior circulation, such as ICA and middle cerebral artery (MCA), particularly the M1 and M2 segments (Online Supplementary Table 2).

**Table 1:** Main characteristics of included studies.

**Reference**	**Publication time**	**Included patients (*n*)**	**FPE (*n*, %)**	**Recruit period**	**Recanalization**	**Location**	**Center**
Velasco Gonzalez et al. (20)	2020	200	102, 51.0	2016.1–2018.12	Complete	Europe	Single
Srivatsa et al. (19)	2020	76	35, 46.1	2016–2018	Complete	North America	Single
Mokin M et al. (18)	2020	609	140, 23.0	2013.3–2015.8	Complete	North America	Multiple
Mohammaden et al. (11)	2020	436	254, 58.3	2012.1–2019.5	Complete	North America	Single
Kang DH et al. (9)	2020	344	66, 19.2	2011.1–2015.12	Complete	Asia	Multiple
Ducroux et al. (28)	2020	336	97, 28.9	2015.10–2016.10	Complete	Europe	Multiple
Di Maria et al. (17)	2020	1832	417, 22.8	2013.10–2018.4	Complete	Europe	Multiple
García-Tornel et al. (10)	2020	459	213, 46.4	2012–2019	Successful	Europe	Single
Yi et al. (16)	2019	61	25, 41.0	2015.1–2016.10	Successful	Asia	Single
Tomasello et al. (15)	2019	193	97, 50.3	2017.2–2017.6	Successful	Europe	Multiple
Nikoubashman et al. (29)	2019	164	62, 37.8	2010.5–2018.1	Complete	Europe	Single
Anadani et al. (8)	2019	524	178, 34.0	2013.11–2018.1	Complete	North America	Multiple
Zaidat et al. (7)	2018	345	89, 25.8	2012.3–2013.2	Complete	North America	Multiple
Imahori et al. (30)	2018	50	21, 42.0	2015.7–2017.6	Successful	Asia	Single
Flottmann et al. (31)	2018	330	151, 46	2019–2017	Successful	Europe	Single
Baek et al. (13)	2017	136	68, 50.0	2010.9–2015,8	Successful	Asia	Single

*FPE, first-pass effect*.

### Influencing Factors

The following factors were assessed: age, gender, hypertension, DM, coronary artery disease, smoking history, atrial fibrillation, dyslipidemia, previous anticoagulation therapy, initial NIHSS score, systolic blood pressure, diastolic blood pressure, suspected stroke etiology, IV thrombolysis, stroke laterality, location of occlusion, anterior communicating artery (AComA) and posterior communicating artery (PComA) presence, and intervention characteristics.

### Determinants for Achieving Successful Recanalization With FPE

**Figure 2:**
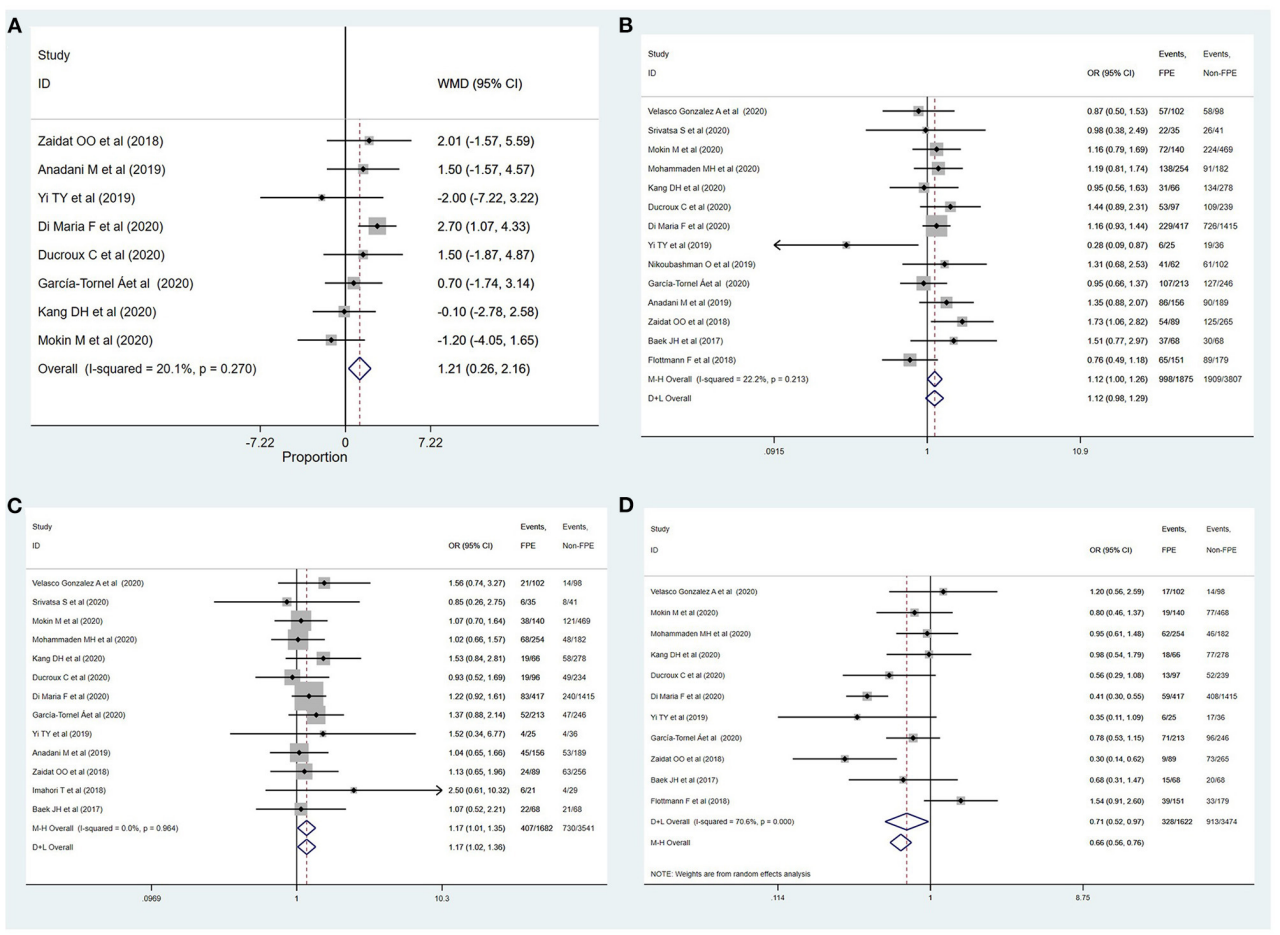
Determinants of achieving successful recanalization with FPE. **(A)** Age; **(B)** female; **(C)** diabetes mellitus; **(D)** ICA.

**Figure 3:**
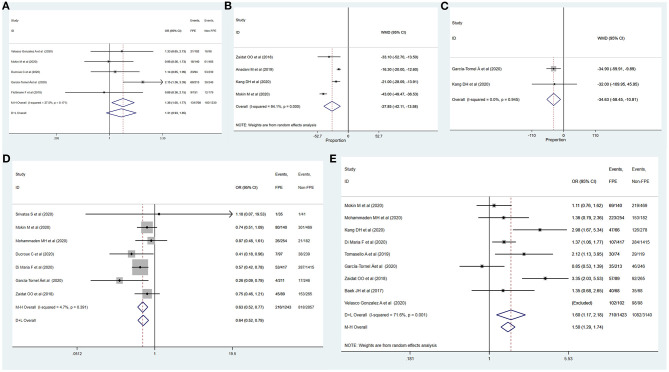
Determinants of achieving successful recanalization with FPE. **(A)** M2; **(B)** duration of intervention; **(C)** time of onset to recanalization; **(D)** general anesthesia; **(E)** use of BGC.

The outcomes of meta-analysis showed that age (MD: 1.21, 95% CI: 0.26–2.16; *p* = 0.012), female gender (OR: 1.12, 95% CI: 1.00–1.26; *p* = 0.046), DM (OR: 1.17, 95% CI: 1.01–1.35; *p* = 0.032), ICA location (OR: 0.71, 95% CI: 0.52–0.97; *p* = 0.033), M2 segment (OR: 1.36, 95% CI: 1.05–1.77; *p* = 0.019), duration of intervention (MD: −27.85, 95% CI: −42.11–13.58; *p* < 0.001), time of onset to recanalization (MD: −34.63, 95% CI: −58.45–10.81; *p* = 0.004), general anesthesia (OR: 0.63, 95% CI: 0.52–0.77; *p* < 0.001), and use of BGC (OR: 1.60, 95% CI: 1.17–2.18; *p* = 0.003) were significantly associated with successful recanalization with FPE (Table 2 and Figures 2, 3). The remainder were not significantly correlated with achieving successful recanalization with FPE (online Supplementary Figures 1, 2).

**Table 2:** Summary of meta-analysis of influencing factors for achieving successful recanalization with FPE.

	**WMD/OR**	**95%CI**		***I*^**2**^ (%)**	***P*-value**
Age	1.21	0.26	2.16	20.1	**0.012**
Gender, female	1.12	1.00	1.26	22.2	**0.046**
Hypertension	1.10	0.97	1.26	43.1	0.134
Diabetes mellitus	1.17	1.01	1.35	0.0	**0.032**
CAD	0.88	0.69	1.13	0.0	0.320
Smoke	0.93	0.80	1.09	7.4	0.364
Atrial fibrillation	0.96	0.67	1.38	77.2	0.836
Previous anticoagulation therapy	1.16	0.93	1.45	20.6	0.197
Dyslipidemia	1.08	0.95	1.23	0.0	0.227
Initial NIHSS score	−0.65	−1.34	0.05	0.0	0.067
Systolic blood pressure	−1.31	−3.41	0.80	17.0	0.223
Diastolic blood pressure	−0.66	−2.12	0.79	42.0	0.374
Suspected stroke etiology					
Large artery atherosclerosis	1.07	0.76	1.51	0.0	0.710
Cardioembolic	1.01	0.60	1.70	72.3	0.973
Other	1.13	0.92	1.38	51.8	0.240
IV thrombolysis	1.015	0.904	1.141	0.0	0.798
Stroke demographics					
Laterality					
Right	0.56	0.14	2.15	93.4	0.398
Left	1.24	0.21	7.27	90.2	0.812
Location of occlusion					
ICA	0.71	0.52	0.97	70.6	**0.033**
M1	1.25	0.88	1.77	81.5	0.206
M2	1.36	1.05	1.77	37.5	**0.019**
Tandem occlusion	0.86	0.34	2.13	0.0	0.737
Ipsilateral AComA and PComA	0.73	0.42	1.24	34.7	0.243
Intervention characteristics					
Time of onset to puncture	20.42	0.00	40.83	0.0	0.050
Duration of intervention	−27.85	−42.11	−13.58	94.1	**<0.001**
Time of onset to recanalization	−34.63	−58.45	−10.81	0.0	**0.004**
General anesthesia	0.63	0.52	0.77	4.7	**<0.001**
Aspiration only	1.31	0.62	2.76	81.9	0.481
Stent retriever only	1.07	0.58	1.96	78.3	0.839
Aspiration and stent retriever both	0.59	0.20	1.72	90.3	0.334
Use of BGC	1.60	1.17	2.18	71.6	**0.003**
Migration to new territory	0.55	0.25	1.22	0.0	0.142

*FPE, first-pass effect; WMD, weighted mean difference; OR, odds ratio; CI, confidence interval; I^2^, the variation attributable to heterogeneity, CAD, coronary artery disease; NIHSS, National Institutes of Health Stroke Scale; IV, intravenous; ICA, internal carotid artery; M1, M1 segment of middle cerebral artery; M2, M2 segment of middle cerebral artery; AComA, anterior communicating artery; PComA, posterior communicating artery; BGC, balloon-guided catheter. Bold values mean P-value with significant difference*.

### Determinants for Achieving Complete Recanalization With FPE

**Figure 4:**
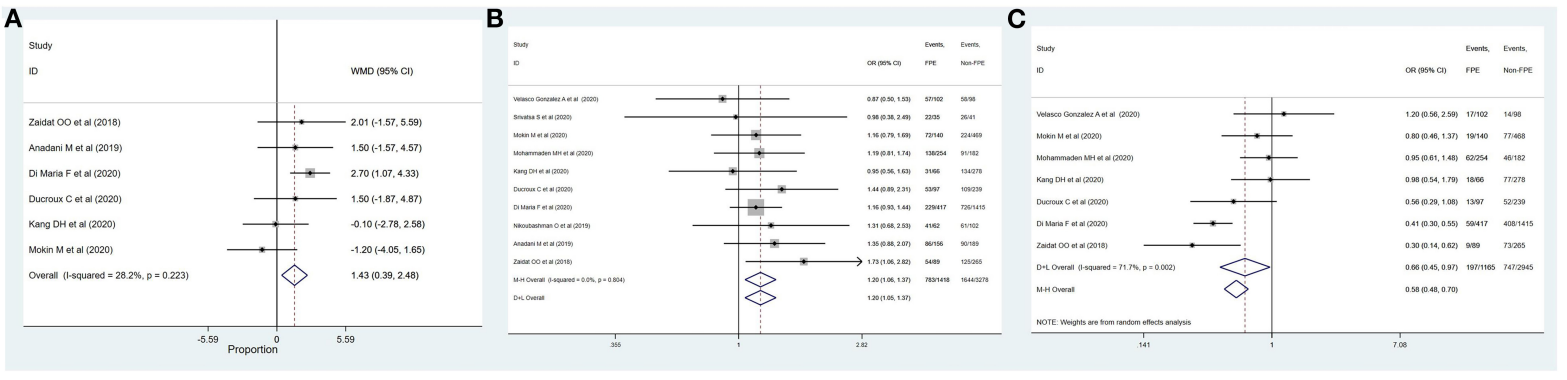
Determinants of achieving complete recanalization with FPE. **(A)** Age; **(B)** female; **(C)** ICA.

**Figure 5:**
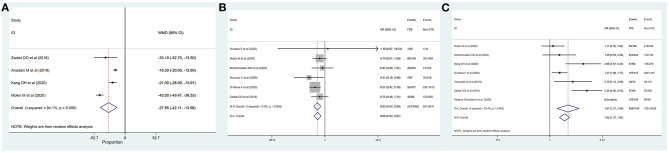
Determinants of achieving complete recanalization with FPE. **(A)** Duration of intervention; **(B)** general anesthesia; **(C)** use of BGC.

Table 3 summarizes the results of meta-analysis of factors influencing complete recanalization with FPE. Age (MD: 1.43, 95% CI: 0.39–2.48; *p* = 0.007), female gender (OR: 1.20, 95% CI: 1.06–1.37; *p* = 0.006), ICA (OR: 0.66, 95% CI: 0.45–0.97; *p* = 0.035), duration of intervention (MD: −27.85, 95% CI: −42.11–13.58; *p* < 0.001), general anesthesia (OR: 0.65, 95% CI: 0.54–0.80; *p* < 0.001), and use of BGC (OR: 1.81, 95% CI: 1.27–2.59; *p* = 0.001) were significantly associated with the complete recanalization with FPE (Figures 4, 5). The remainder were not significantly correlated with achieving successful recanalization with FPE (Online Supplementary Figures 3, 4).

**Table 3:** Summary of meta-analysis of influencing factors for achieving complete recanalization with FPE.

	**WMD/OR**	**95% CI**		***I*^**2**^ (%)**	***P*-value**
Age	1.43	0.39	2.48	28.2%	**0.007**
Gender, female	1.20	1.06	1.37	0.0%	**0.006**
Hypertension	1.06	0.92	1.22	37.1%	0.423
Diabetes mellitus	1.14	0.97	1.34	0.0%	0.106
CAD	0.81	0.61	1.07	0.0%	0.131
Smoke	0.96	0.80	1.14	0.0%	0.605
Atrial fibrillation	0.97	0.81	1.17	61.8%	0.747
Previous anticoagulation therapy	1.16	0.93	1.45	20.6%	0.197
Dyslipidemia	1.08	0.94	1.24	0.0%	0.286
Initial NIHSS score	−0.54	−1.26	0.17	0.0%	0.135
Systolic blood pressure	−1.91	−4.25	0.43	11.2%	0.109
Diastolic blood pressure	−1.53	−3.26	0.19	0.0%	0.081
Suspected stroke etiology					
Large artery atherosclerosis	1.09	0.77	1.56	0.0%	0.617
Cardioembolic	1.01	0.60	1.70	72.3%	0.973
Other	1.12	0.92	1.38	67.6%	0.261
IV thrombolysis	0.99	0.87	1.13	0.0%	0.940
Stroke demographics					
Laterality					
Right	0.81	0.14	4.73	90.2%	0.812
Left	1.24	0.21	7.27	90.2%	0.812
Location of occlusion					
ICA	0.66	0.45	0.97	71.7%	**0.035**
M1	1.44	0.97	2.15	80.4%	0.072
M2	1.12	0.79	1.58	0.0%	0.534
Tandem occlusion	0.86	0.34	2.13	0.0%	0.737
Ipsilateral AComA and PComA	0.73	0.42	1.24	34.7%	0.243
Intervention characteristics					
Time of onset to puncture	11.36	−23.38	46.09	9.0	0.522
Duration of intervention	−27.85	−42.11	−13.58	94.1	**<0.001**
General anesthesia	0.65	0.54	0.80	0.0%	**<0.001**
Aspiration only	1.48	0.69	3.16	85.2%	0.312
Stent retriever only	0.90	0.55	1.48	71.7%	0.689
Aspiration and stent retriever both	0.67	0.22	2.04	93.1%	0.479
Use of BGC	1.81	1.27	2.59	73.4%	**0.001**
Migration to new territory	0.55	0.25	1.22	0.0%	0.142

*FPE, first-pass effect; WMD, weighted mean difference; OR, odds ratio; CI, confidence interval; I^2^, the variation attributable to heterogeneity; CAD, coronary artery disease; NIHSS, National Institutes of Health Stroke Scale; IV, intravenous; ICA, internal carotid artery; M1, M1 segment of middle cerebral artery; M2, M2 segment of middle cerebral artery; AComA, anterior communicating artery; PComA, posterior communicating artery; BGC, balloon-guided catheter. Bold values mean P-value with significant difference*.

### Risk of Bias in Studies Included

The Newcastle–Ottawa scale was used to assess the bias risk of observational studies, such as case–control studies, with the majority of included studies being low risk bias (online Supplementary Table 3). Both meta-regression and sensitive analysis were conducted to explore the potential heterogeneity. We also used funnel plots to explore the publication bias, with the results demonstrating no evident reporting bias (Supplementary Figures 5–16).

## Discussion

In this systematic review and meta-analysis, the proportion of FPE ranged from 19 to 58% in the endovascular treatment of LVO inclusive of M2 occlusions. Factors contributing to successful recanalization with FPE included age, female gender, DM, general anesthesia, use of BGC, and occlusion of ICA and M2 segment. Among those, age, female gender, general anesthesia, use of BGC, and occlusion of ICA also increased the chance of complete reperfusion after first-pass thrombectomy.

BGC use has been widely accepted contributing to FPE during thrombectomy procedure (7, 9, 13, 15). One of the reasons may be decreased distal embolization and more importantly increased flow reversal. According to Kang et al. (9), additional positive effects of BGC use were suggested. One is the force needed for clot retrieving, including impaction force and combined force of friction and adhesion between the thrombus and vessel wall. The other is that inflating the BGC can markedly reduce systemic blood pressure on the proximal clot surface and decrease the pressure gradient across the clot.

It is difficult to explain why increased age was found a contributor to successful and complete recanalization after first-pass thrombectomy. One possible reason may be stroke etiology, as elderly patients are more likely to have cardioembolic cause (32). Clots from cardioembolic causes are more likely to be rich in red blood cells whereas thromboembolism due to preexisting atherosclerosis may be rich in fibrin and platelets. Clots composed predominantly of RBCs are considered fresh and less compact, and this may lead to easier recanalization through thrombectomy with first pass (33–37). Also, increased fibrin percentage could decrease the possibility of clot complete retrieval (38, 39). However, heterogeneous results exist among studies and we could not detect a relationship between FPE and stroke etiology, which may be due to limited data.

This study showed that females are more likely to achieve FPE than males. This phenomenon has been described by Zaidat et al. (7). Anatomical, pathophysiological, and biochemical factors may potentially account for observed difference in response to recanalization therapy between sexes (40). In addition, there was a difference of endogenous fibrinolytic activity between males and females (35, 41). Further studies are needed to explore the underlying biochemical interactions which are further felt to change with age/menopause (42).

Conscious sedation has been associated with better outcomes of MT than general anesthesia in previous researches (43). In this study, we further extended this preference of local anesthesia considering FPE, but the mechanisms remain unknown (17). One hypothesis is a shorter time to reperfusion by conscious sedation (44), as dynamic changes of clot composition found in previous studies, such as fibrin deposition, may increase the risk of re-occlusion (34, 36). However, some studies have mentioned that GA is associated with better outcome than conscious sedation. So, comparison of different anesthesia modalities needs further research (45). At the same time, difference in clot length may be a principal reason for the association between clot location and FPE. It was found that clots in ICA were with longer length and those in M2 segment were with relatively shorter length (7, 17).

There are some limitations of this study. Recruited studies were mostly retrospective with small sample size, and variables observed were not uniform. Some potentially important factors, such as clot volume (13), were only investigated occasionally and thus could not be reliably meta-analyzed. Potential differences may exist between anterior and posterior circulation stroke, and separate analysis may be more valuable. Also, device development could also influence the recanalization outcome, and comparison among different thrombectomy techniques may also be very important. However, it is unable to be analyzed due to high heterogeneity among studies. It remains elusive why DM was a contributor to successful recanalization with FPE (46). Maybe this is caused by bias from limited studies and should be further studied.

## Conclusion

Age, gender, occlusion site, conscious sedation, and use of BGC were factors influencing both successful and complete recanalization after first-pass thrombectomy. Further studies with more comprehensive observational indices are needed to confirm these observations.

## Data Availability Statement

The original contributions presented in the study are included in the article/Supplementary Material, further inquiries can be directed to the corresponding author/s.

## Author Contributions

XB, XZ, and LJ developed the initial idea for this study and formulated the study design. YZ, AD, TW, RX, YF, XW, and KY developed and revised the search strategy. LJ, YM, HS, and QM were consulted about clinical issues. XB, XZ, and JW contributed to the original draft. XB, XZ, AD, JW, YZ, TW, LL, KY, YM, HS, QM, and LJ were responsible for the revision of the draft. All authors approved the final version of the manuscript before submission.

## Conflict of Interest

The authors declare that the research was conducted in the absence of any commercial or financial relationships that could be construed as a potential conflict of interest.
