# Supplementary material for: Factors Influencing Recanalization After Mechanical Thrombectomy With First-Pass Effect for Acute Ischemic Stroke: A Systematic Review and Meta-Analysis
Source: Front Neurol. 2021 Apr 9;12:628523. doi: 10.3389/fneur.2021.628523 (PMC8062801; doi:10.3389/fneur.2021.628523)
Supplement: Supplementary file 1 [file Data_Sheet_1.docx]

**Supplementary Table 1** Search strategy for Medline

| Search step | Numbers | Search terms |
| --- | --- | --- |
| #1 | 596,930 | (((("stroke"[MeSH Terms] OR "stroke"[All Fields]) OR "strokes"[All Fields]) OR "stroke s"[All Fields]) OR (((("cerebral infarction"[MeSH Terms] OR ("cerebral"[All Fields] AND "infarction"[All Fields])) OR "cerebral infarction"[All Fields]) OR ("cerebral"[All Fields] AND "infarct"[All Fields])) OR "cerebral infarct"[All Fields])) OR ((((((((("infarctation"[All Fields] OR "infarcted"[All Fields]) OR "infarctic"[All Fields]) OR "infarcting"[All Fields]) OR "infarction"[MeSH Terms]) OR "infarction"[All Fields]) OR "infarct"[All Fields]) OR "infarctions"[All Fields]) OR "infarcts"[All Fields]) OR "infarctive"[All Fields]) |
| #2 | 59, 004 | ((("thrombectomy"[MeSH Terms] OR "thrombectomy"[All Fields]) OR "thrombectomies"[All Fields]) OR (((((("mechanical"[All Fields] OR "mechanically"[All Fields]) OR "mechanicals"[All Fields]) OR "mechanics"[MeSH Terms]) OR "mechanics"[All Fields]) OR "mechanic"[All Fields]) AND (("thrombectomy"[MeSH Terms] OR "thrombectomy"[All Fields]) OR "thrombectomies"[All Fields]))) OR ("endovascular"[All Fields] AND (((((("therapeutics"[MeSH Terms] OR "therapeutics"[All Fields]) OR "therapies"[All Fields]) OR "therapy"[MeSH Subheading]) OR "therapy"[All Fields]) OR "therapy s"[All Fields]) OR "therapys"[All Fields])) |
| #3 | 72, 963 | (("first"[All Fields] OR "firsts"[All Fields]) AND "Pass"[All Fields]) OR (("first"[All Fields] OR "firsts"[All Fields]) AND ((((("attempt"[All Fields] OR "attempted"[All Fields]) OR "attempter"[All Fields]) OR "attempters"[All Fields]) OR "attempting"[All Fields]) OR "attempts"[All Fields])) |
| #4 | 283 | #1 AND #2 AND #3 |

**Supplementary Table 2** Baseline characteristics of patients in included studies

| Study | Velasco Gonzalez A et al 2020 | | Srivatsa S et al 2020 | | Mokin M et al 2020 | | Mohammaden MH et al 2020 | | Kang DH et al 2020 | | Ducroux C et al 2020 | | Di Maria F et al 2020 | |
| --- | --- | --- | --- | --- | --- | --- | --- | --- | --- | --- | --- | --- | --- | --- |
|  | Yes (102) | No (98) | Yea (35) | No (41) | Yea (140) | No (469) | Yea (254) | No (182) | Yes (66) | No (278) | Yes (97) | No (239) | Yes (417) | No (1415) |
| Age, years, Mean ± SD, Median [IQR] | 76 (68, 81) | 78 (71,85) | 65.7 (60.4, 71.0) | 66.5 (61.9, 71.2) | 65.3±15.3 | 66.5±14.5 | NR | NR | 68.5 ±9,6 | 68.6±11.4 | 70.5±14.0 | 69.0±14.9 | 71.9±14.6 | 69.2±15.8 |
| Gender, female, n (%) | 57 (55.9) | 58 (59.2) | 22 (62.9) | 26 (63.4) | 72 (51) | 224 (48) | 138 (54.3) | 91 (50) | 31 (47) | 134 (48.2) | 53 (54.6) | 109 (45.6) | 229 (54.9) | 726 (51.3) |
| Hypertension, n (%) | 55 (53.9) | 65 (66.3) | 27(77.1) | 21 (51.2) | 103 (74) | 357 (76) | 194 (76.4) | 133 (73.1) | 41 (62.1) | 177 (63.7) | 53/95 (55.8) | 145/235 (61.7) | 260 (62.4) | 826 (58.4) |
| Diabetes mellitus, n (%) | 21 (20.6) | 14 (13.7) | 6 (17.1) | 8 (19.5) | 38 (27) | 121 (26) | 68 (26.8) | 48 (26.4) | 19 (28.8) | 58 (20.9) | 19/96 (19.8) | 49/234 (20.9) | 83 (20.0) | 240 (17.0) |
| CAD, n (%) | NR | NR | 9 (25.7) | 16 (39) | 26/107 (24) | 112/374 (30) | NR | NR | 9 (13.6) | 36 (12.9) | 14/95 (14.8) | 45/232 (19.4) | NR | NR |
| Smoke, n (%) | 14 (13.7) | 10 (10.2) | 11 (31.4) | 10 (24.4) | 28 (20) | 124/466 (27) | 43 (16.9) | 24 (13.2) | 24 (36.4) | 88 (31.7) | 16/76 (21.1) | 41/206 (19.9) | 79 (19) | 298 (21.1) |
| Atrial fibrillation, n (%) | 54 (53) | 45 (45.9) | NR | NR | 57/139 (41) | 179 (38) | 85 (33.5) | 88 (48.4) | 40 (60.6) | 145 (52.2) | NR | NR | NR | NR |
| Previous anticoagulation therapy, n (%) | 46 (45.1) | 39 (39.8) | NR | NR | NR | NR | NR | NR | NR | NR | 15/93 (16.1) | 50/237 (21.1) | 97 (23.3) | 277 (19.6) |
| Antiplatelet drugs, n (%) | NR | NR | NR | NR | NR | NR | NR | NR | NR | NR | NR | NR | 104 (24.9) | 341 (24.1) |
| Dyslipidemia , n (%) | 21 (21.4) | 21 (21.4) | NR | NR | 76 (54) | 229 (49) | 81 (31.9) | 64 (35.2) | 20 (30.3) | 85 (30.6) | 34/96 (35.4) | 86/232 (37.1) | 260 (62.4) | 826 (58.4) |
| Suspected stroke cause | | | | | | | | | | | | | | |
| Large artery atherosclerosis , n (%) | NR | NR | NR | NR | NR | NR | NR | NR | NR | NR | 10/97 (10.3) | 18/239 (7.5) | 32 (7.6) | 105 (7.4) |
| Cardioembolic, n (%) | NR | NR | NR | NR | NR | NR | NR | NR | NR | NR | 33/97 (34.0) | 109/239 (45.6) | 250 (60.0) | 849 (60.0) |
| Other, n (%) | NR | NR | NR | NR | NR | NR | NR | NR | NR | NR | 54/97 (55.7) | 112/239 (46.9) | 135 (32.3) | 461 (32.6) |
| Stroke demographics | | | | | | | | | | | | | | |
| Laterality | | | | | | | | | | | | | | |
| Right, n (%) | 60 (58.8) | 42 (41.2) | 10 (28.6) | 23 (56.1) | NR | NR | NR | NR | NR | NR | NR | NR | NR | NR |
| Left, n (%) | 42 (42.9) | 56 (57.1) | 25 (71.4) | 18 (43.9) | NR | NR | NR | NR | NR | NR | NR | NR | NR | NR |
| IV thrombolysis, n (%) | 55 (53.9) | 54 (55.1) | 22 (62.9) | 22 (53.7) | 66/138 (48) | 241/467 (52) | 92 (36.2) | 73 (40.1) | 29 (43.9) | 143 (51.4) | 61/97 (62.9) | 155/239 (64.9) | 245 (58.8) | 814 (57.5) |
| Location of occlusion | | | | | | | | | | | | | | |
| ICA, n (%) | 17 (16.7) | 14 (14.3) | NR | NR | 19/140 (14) | 77/468 (16) | 62 (24.4) | 46 (25.3) | 18 (27.3) | 77 (27.7) | 13/97 (13.4) | 52/239 (21.8) | 59 (14.1) | 408 (28.8) |
| M1, n (%) | 70 (68.7) | 62 (63.3) | NR | NR | 72/140 (51) | 261/468 (56) | 192 (75.6) | 136 (74.7) | NR | NR | 61/97 (62.9) | 134/239 (56.1) | 357 (85.6) | 994 (70.2) |
| M2, n (%) | 21 (20.8) | 16 (16.3) | NR | NR | 18/140 (13) | 61/468 (13) | NR | NR | NR | NR | 23/97 (23.7) | 53/239 (22.2) | NR | NR |
| BA, n (%) | NR | NR | NR | NR | 24/140 (17) | 39/468 (8) | NR | NR | NR | NR | NR | NR | NR | NR |
| VA, n (%) | NR | NR | NR | NR | 3/140 (2) | 12/468 (3) | NR | NR | NR | NR | NR | NR | NR | NR |
| Tandem occlusion n (%) | NR | NR | NR | NR | 1/140 (0.7) | 5/468 (1) | NR | NR | 5 (7.6) | 23 (8.3) | NR | NR | NR | NR |
| Systolic blood pressure, Mean ± SD, Median [IQR] | NR | NR | NR | NR | NR | NR | NR | NR | NR | NR | 148±25 | 146±26 | 143.6±24.7 | 146.5±25.9 |
| Diastolic blood pressure , Mean ± SD, Median [IQR] | NR | NR | NR | NR | NR | NR | NR | NR | NR | NR | NR | NR | 79.7±17.3 | 81.3±17.3 |
| Initial median NIHSS score , Mean ± SD, Median [IQR] | 15 (11-17) | 15 (11-17) | 16 (2-28) | 16 (3-26) | 17.1±6.9 | 17.4±6.6 | NR | NR | 15±8 | 15±8 | 15.8±6.5 | 16.4±6.0 | 17 (12-21) | 18 (13-21) |
| Final median NIHSS score, Mean ± SD, Median [IQR] | 3 (2-7) | 4 (2-7) | 3 (0-23) | 11 (1-28) | 9.1±11.2 | 12±11 | NR | NR | NR | NR | NR | NR | NR | NR |
| ASPECTS, Mean ± SD, Median [IQR] | NR | NR | 10 (7-10) | 10 (6-10) | NR | NR | NR | NR | 8±2 | 8±1 | 8 (6-9) | 7 (5-9) | 8 (6-9) | 7 (5-8) |
| Pre-stroke mRS 0-1, n (%) | NR | NR | NR | NR | 108/130 (83) | 387/431 (90) | NR | NR | NR | NR | NR | NR | NR | NR |
| Pre-stroke mRS ≥1, n (%) | NR | NR | NR | NR | NR | NR | NR | NR | NR | NR | 24/96 (25.0) | 33/239 (13.8) | NR | NR |
| Retrieval conditions | | | | | | | | | | | | | | |
| Ipsilateral AComA and PComA, n (%) | 18 (40) | 27 (60) | 20 (57.1) | 22 (53.7) | NR | NR | NR | NR | NR | NR | NR | NR | NR | NR |
| Elongation, n (%) | 27 (26.5) | 56 (57.1) | NR | NR | NR | NR | NR | NR | NR | NR | NR | NR | NR | NR |
| Intervention characteristics | | | | | | | | | | | | | | |
| Onset to puncture(min), Mean ± SD, Median [IQR] | NR | NR | NR | NR | 384.4±352.9 | 358.1±234.8 | NR | NR | NR | NR | 230 (179-283) | 230 (184-279) | 240 (195-305) | 243 (186-305) |
| Duration of intervention(min), Mean ± SD, Median [IQR] | 16 (14-18) | 35 (22-59) | 28.9 (25.1-32.7) | 46.8 (37.8-55.7) | 46.3±27.4 | 89.3±51 | NR | NR | 34±22 | 55±40 | NR | NR | NR | NR |
| Onset to recanalization(min), Mean ± SD, Median [IQR] | NR | NR | NR | NR | NR | NR | NR | NR | 288±301 | 320±241 | NR | NR | NR | NR |
| General anesthesia, n (%) | NR | NR | 1 (2.9) | 1 (2.4) | 80 (57) | 301 (64) | 26 (10.2) | 21 (11.5) | NR | NR | 7/97 (7.2) | 38/239 (15.9) | 53 (12.7) | 287 (20.3) |
| Aspiration only, n (%) | NR | NR | 24 (68.6) | 6 (14.6) | NR | NR | 30 (11.8) | 22 (12.1) | NR | NR | NR | NR | 212 (50.8) | 731 (51.7) |
| Stent retriever only, n (%) | NR | NR | 6 (17.1) | 9 (22) | NR | NR | 176 (69.3) | 118 (64.8) | NR | NR | NR | NR | 93 (22.3) | 457 (32.3) |
| Aspiration and stent retriever both, n (%) | NR | NR | 5 (14.3) | 23 (56.1) | NR | NR | 48 (18.9) | 42 (23.1) | NR | NR | NR | NR | 112 (26.9) | 227 (16.0) |
| Use of BGC, n (%) | 102 (100) | 98 (100) | NR | NR | 69 (49) | 219 (47) | 223 (87.8) | 153 (84.1) | 47 (71.2) | 126 (45.3) | NR | NR | 107 (25.7) | 284 (20.1) |
| BGC position, n (%) | | | | | | | | | | | | | | |
| Distal ICA, n (%) | 70 (68.6) | 39 (39.8) | NR | NR | NR | NR | NR | NR | NR | NR | NR | NR | NR | NR |
| Proximal ICA, n (%) | 31 (30.4) | 51 (52) | NR | NR | NR | NR | NR | NR | NR | NR | NR | NR | NR | NR |
| Distal common carotid artery, n (%) | 1 (1) | 8 (8.2) | NR | NR | NR | NR | NR | NR | NR | NR | NR | NR | NR | NR |
| Migration to new territory, n (%) | 2 (2) | 4 (4.1) | NR | NR | 4/95 (4) | 16/334 (5) | NR | NR | NR | NR | NR | NR | NR | NR |

| Study | Yi TY et al 2019 | | Tomasello A et al 2019 | | Nikoubashman O et al 2019 | | García-Tornel Áet al 2020 | | Anadani M et al 2019 | | Zaidat OO et al 2018 | | Imahori T et al 2018 | | Flottmann F 2018 | | Baek JH et al 2017 | |
| --- | --- | --- | --- | --- | --- | --- | --- | --- | --- | --- | --- | --- | --- | --- | --- | --- | --- | --- |
|  | Yes (25) | No (36) | Yes (74) | No (119) | Yes (62) | No (102) | Yes (213) | No (246) | Yes (156) | No (189) | Yes (89) | No (265) | Yes (21) | No (29) | Yes (151) | No (179) | Yes (68) | No (97) |
| Age, year, Mean ± SD, Median [IQR] | 62±11 | 64±9 | NR | NR | 77 (67-82) | 75 (63-83) | 72±13 | 71.3±13.6 | 67.8±14.5 | 66.3±14.5 | 68.8±14.8 | 66.79±15.3 | 81 (75-85) | 76 (71-83) | 68±15 | NR | 69.4±11.5 | NR |
| Gender, female, n (%) | 6 (24.0) | 19 (52.8) | NR | NR | 41 (66) | 61 (60) | 107 (50.2) | 127 (51.6) | 86 (55) | 90 (48) | 54 (60.7) | 125 (47.2) | NR | NR | 65 (43) | 89 (49.7) | 37 (54.4) | 46 (47.4) |
| Hypertension, n (%) | 18 (80.0) | 16 (44.4) | NR | NR | NR | NR | 156 (73.3) | 164 (66.7) | 117 (75) | 140 (74) | 71 (79.8) | 200 (75.5) | 16 (76) | 16 (55) | NR | NR | 45 (66.2) | 73 (75.3) |
| Diabetes mellitus, n (%) | 4 (16.0) | 4 (11.1) | NR | NR | NR | NR | 52 (24.4) | 47 (19.1) | 45 (29) | 53 (28) | 24 (27.0) | 63 (23.8) | 6 (29) | 4 (14) | NR | NR | 22 (32.4) | 30 (30.9) |
| CAD, n (%) | NR | NR | NR | NR | NR | NR | 38 (17.8) | 38 (15.4) | c | NR | 27 (30.3) | 84 (31.7) | NR | NR | NR | NR | NR | NR |
| Smoke, n (%) | 16 (64.0) | 16 (44.4) | NR | NR | NR | NR | 50 (23) | 75 (30.4) | NR | NR | 25 (28.1) | 83 (31.3) | NR | NR | NR | NR | 10 (14.7) | 13 (13.4) |
| Atrial fibrillation, n (%) | 3 (12.0) | 28 (77.8) | NR | NR | NR | NR | 76 (35.7) | 69 (28) | 46 (29) | 59 (31) | 40 (44.9) | 108 (40.8) | 15 (71) | 13 (45) | NR | NR | 41 (60.3) | 63 (64.9) |
| Previous anticoagulation therapy, n (%) | NR | NR | NR | NR | NR | NR | NR | NR | NR | NR | NR | NR | NR | NR | NR | NR | NR | NR |
| Antiplatelet drugs, n (%) | NR | NR | NR | NR | NR | NR | NR | NR | NR | NR | NR | NR | NR | NR | NR | NR | NR | NR |
| Dyslipidemia, n (%) | 4 (16.0) | 3 (8.3) | NR | NR | NR | NR | 92 (43.2) | 111(45.1) | 67 (43) | 87 (46) | 50 (56.2) | 132 (49.8) | 4 (19) | 3 (10) | NR | NR | 16 (23.5) | 14 (14.4) |
| Suspected stroke cause | | | | | | | | | | | | | | | | | | |
| Large artery atherosclerosis, n (%) | NR | NR | NR | NR | 3 (5) | 5 (5) | NR | NR | NR | NR | NR | NR | 4(19) | 7(24) | NR | NR | NR | NR |
| Cardioembolic, n (%) | NR | NR | NR | NR | 53 (85) | 74 (73) | NR | NR | NR | NR | NR | NR | NR | NR | NR | NR | NR | NR |
| Other, n (%) | NR | NR | NR | NR | 6 (10) | 23 (23) | NR | NR | NR | NR | NR | NR | 17 (81) | 22 (76) | NR | NR | NR | NR |
| Stroke demographics | | | | | | | | | | | | | | | | | | |
| Laterality | | | | | | | | | | | | | | | | | | |
| Right, n (%) | NR | NR | NR | NR | NR | NR | 113 (53.1) | 197 (43.9) | NR | NR | NR | NR | NR | NR | NR | NR | NR | NR |
| Left, n (%) | NR | NR | NR | NR | NR | NR | NR | NR | NR | NR | NR | NR | NR | NR | NR | NR | NR | NR |
| IV thrombolysis, n (%) | 8 (32.0) | 9 (25.0) | NR | NR | 45 (73) | 71 (70) | 98 (46) | 116 (47.2) | 73 (47) | 76 (40) | 39 (43.8) | 115 (43.4) | 2 (10) | 6 (21) | 98 (65) | 104 (58.1) | 29 (42.7) | 35 (36.1) |
| Location of occlusion | | | | | | | | | | | | | | | | | | |
| ICA, n (%) | 6 (24.0) | 17 (47.2) | NR | NR | NR | NR | 71 (35.2) | 96 (39.0) | NR | NR | 9 (10.1) | 73 (27.1) | NR | NR | 39 (26) | 33 (18.4) | 15 (22.1) | 31 (32.0) |
| M1, n (%) | 16 (64.0) | 18 (44.4) | NR | NR | NR | NR | 120 (56.3) | 150 (61) | NR | NR | 57 (64.0) | 139 (52.5) | NR | NR | 76 (50) | 74 (41.3) | NR | NR |
| M2, n (%) | NR | NR | NR | NR | NR | NR | 60 (28.2) | 38 (15.4) | NR | NR | NR | NR | NR | NR | 9 (6) | 12 (6.7) | NR | NR |
| BA, n (%) | 3 (12.0) | 4 (11.1) | NR | NR | NR | NR | NR | NR | NR | NR | NR | NR | NR | NR | NR | NR | NR | NR |
| VA, n (%) | NR | NR | NR | NR | NR | NR | NR | NR | NR | NR | NR | NR | NR | NR | NR | NR | 11 (16.1) | 13 (13.4) |
| VB, n (%) | NR | NR | NR | NR | NR | NR | NR | NR | NR | NR | 12 (13.5) | 24 (9.1) | NR | NR | NR | NR | NR | NR |
| Tandem occlusion, n (%) | NR | NR | NR | NR | NR | NR | NR | NR | NR | NR | NR | NR | NR | NR | NR | NR | NR | NR |
| Systolic blood pressure, Mean ± SD, Median [IQR] | NR | NR | NR | NR | NR | NR | 145.9±26.9 | 144..6±26 | NR | NR | 144.9±29.4 | 145.7±27.6 | NR | NR | NR | NR | NR | NR |
| Diastolic blood pressure, Mean ± SD, Median [IQR] | NR | NR | NR | NR | NR | NR | 80±15.2 | 78.5±14.3 | NR | NR | 77.7±17.5 | 78.9±17.8 | NR | NR | NR | NR | NR | NR |
| Initial median NIHSS score, Mean ± SD, Median [IQR] | 15.9±6 | 18.2±5 | NR | NR | 17 (12-20) | 17 (17-21) | 16 (11-20) | 16 (11-20) | 15.4±7.6 | 16.1±7.6 | 17.3±7.7 | 18.4±6.2 | 13 (11-22) | 15 (7-21) | 15 (7) | NR | NR | NR |
| Final median NIHSS score, Mean ± SD, Median [IQR] | NR | NR | NR | NR | NR | NR | NR | NR | NR | NR | NR | NR | NR | NR | NR | NR | NR | NR |
| ASPECTS, Mean ± SD, Median [IQR] | NR | NR | NR | NR | 10 (8-10) | 9 (8-10) | 9 (9-10) | 9 (8-10) | NR | NR | NR | NR | 9 (8-10) | 9 (7-10) | NR | NR | NR | NR |
| Pre-stroke mRS 0-1, n (%) | NR | NR | NR | NR | NR | NR | NR | NR | NR | NR | NR | NR | NR | NR | NR | NR | NR | NR |
| Pre-stroke mRS ≥1, n (%) | NR | NR | NR | NR | NR | NR | NR | NR | NR | NR | NR | NR | NR | NR | NR | NR | NR | NR |
| Retrieval conditions | | | | | | | | | | | | | | | | | | |
| Ipsilateral AComA and PComA, n (%) | NR | NR | NR | NR | NR | NR | NR | NR | NR | NR | NR | NR | NR | NR | NR | NR | NR | NR |
| Elongation, n (%) | NR | NR | NR | NR | NR | NR | NR | NR | NR | NR | NR | NR | NR | NR | NR | NR | NR | NR |
| Intervention characteristics | | | | | | | | | | | | | | | | | | |
| Onset to puncture, Mean ± SD, Median [IQR] | NR | NR | NR | NR | NR | NR | 237.2±147.3 | 212±125.4 | 456.5±368 | 525.6±718.8 | 369.5±239 | 361.3±239.5 | 230 (98-280) | 126 (76-323) | NR | NR | 190 (150-313) | NR |
| Duration of intervention(min) , Mean ± SD, Median [IQR] | NR | NR | NR | NR | NR | NR | NR | NR | 15.1±9.4 | 31.4±23.8 | 52.5±73.8 | 85.6±101.4 | NR | NR | NR | NR | 45 (34-64.5) | NR |
| Onset to recanalization, Mean ± SD, Median [IQR] | NR | NR | NR | NR | 185 (157-238) | 251 (205-349) | 237.2±147.3 | 272,1±122.5 | NR | NR | NR | NR | NR | NR | NR | NR | NR | NR |
| General anesthesia, n (%) | NR | NR | NR | NR | NR | NR | 4 (1.9) | 17 (6.9) | NR | NR | 45 (50.6) | 153 (57.7) | NR | NR | NR | NR | NR | NR |
| Aspiration only, n (%) | NR | NR | 13 (17.6) | 26 (21.8) | NR | NR | NR | NR | NR | NR | NR | NR | 0 | 4 (14) | NR | NR | NR | NR |
| Stent retriever only, n (%) | NR | NR | 61 (82.4) | 93 (78.2) | NR | NR | NR | NR | NR | NR | NR | NR | 21(100) | 14 (48) | NR | NR | NR | NR |
| Aspiration and stent retriever both, n (%) | NR | NR | NR | NR | NR | NR | NR | NR | NR | NR | NR | NR | 0 | 3 (10) | NR | NR | NR | NR |
| Use of BGC, n (%) | NR | NR | 30 (40.5) | 29 (24.4) | NR | NR | 35 (16.4) | 46 (18.7) | NR | NR | 57 (64.0) | 92 (34.7) | NR | NR | NR | NR | 40 (58.8) | 44 (45.4) |
| BGC position | NR | NR | NR | NR | NR | NR | NR | NR | NR | NR | NR | NR | NR | NR | NR | NR | NR | NR |
| Distal ICA, n (%) | NR | NR | 8 (10.8) | 2 (1.7) | NR | NR | NR | NR | NR | NR | NR | NR | NR | NR | NR | NR | NR | NR |
| Proximal ICA, n (%) | NR | NR | 23 (31.1) | 27 (22.7) | NR | NR | NR | NR | NR | NR | NR | NR | NR | NR | NR | NR | NR | NR |
| Distal common carotid artery, n (%) | NR | NR | NR | NR | NR | NR | NR | NR | NR | NR | NR | NR | NR | NR | NR | NR | NR | NR |
| Migration to new territory, n (%) | NR | NR | NR | NR | NR | NR | NR | NR | NR | NR | 2 (2.3) | 17 (6.4) | NR | NR | NR | NR | NR | NR |

*SD* standard deviation; *IQR* interquartile range; *CAD* coronary artery disease; *IV* intravenous; *ICA* internal carotid artery; *M1* M1 segment of middle cerebral artery; *M2* M2 segment of middle cerebral artery; *BA* basilar artery; *VA* vertebral artery; *VB* vertebrobasilar; *NIHSS* National Institutes of Health Stroke Scale; *ASPECTS* Alberta Stroke Program Early CT Score; *mRS* modified Rankin score; *AComA* anterior communicating artery; *PComA* posterior communicating artery; *BGC* balloon-guided catheter

**Supplementary Table 3** New Castle-Ottawa Scoring for case-control studies

| Study | Subject Selection Max 4 | Study Comparability Max2 | Assessment of Outcomes Max3 | Total Score | Risk of bias |
| --- | --- | --- | --- | --- | --- |
| Velasco Gonzalez A et al | 3 | 2 | 3 | 8 | Low |
| Srivatsa S et al | 3 | 2 | 3 | 8 | Low |
| Mokin M et al | 4 | 2 | 3 | 9 | Low |
| Mohammaden MH et al | 3 | 2 | 3 | 8 | Low |
| Kang DH et al | 4 | 2 | 3 | 9 | Low |
| Ducroux C et al | 4 | 1 | 3 | 8 | Low |
| Di Maria F et al | 3 | 2 | 3 | 8 | Low |
| García-Tornel Á et al | 4 | 2 | 3 | 9 | Low |
| Yi TY et al | 4 | 2 | 2 | 8 | Low |
| Tomasello A et al | 3 | 2 | 3 | 7 | Low |
| Nikoubashman O et al | 4 | 2 | 3 | 9 | low |
| Anadani M et al | 3 | 2 | 3 | 8 | low |
| Zaidat OO et al | 4 | 2 | 3 | 9 | low |
| Imahori T et al | 3 | 2 | 2 | 7 | low |
| Flottmann F et al | 3 | 2 | 2 | 7 | low |
| Baek JH et al | 3 | 2 | 3 | 8 | low |


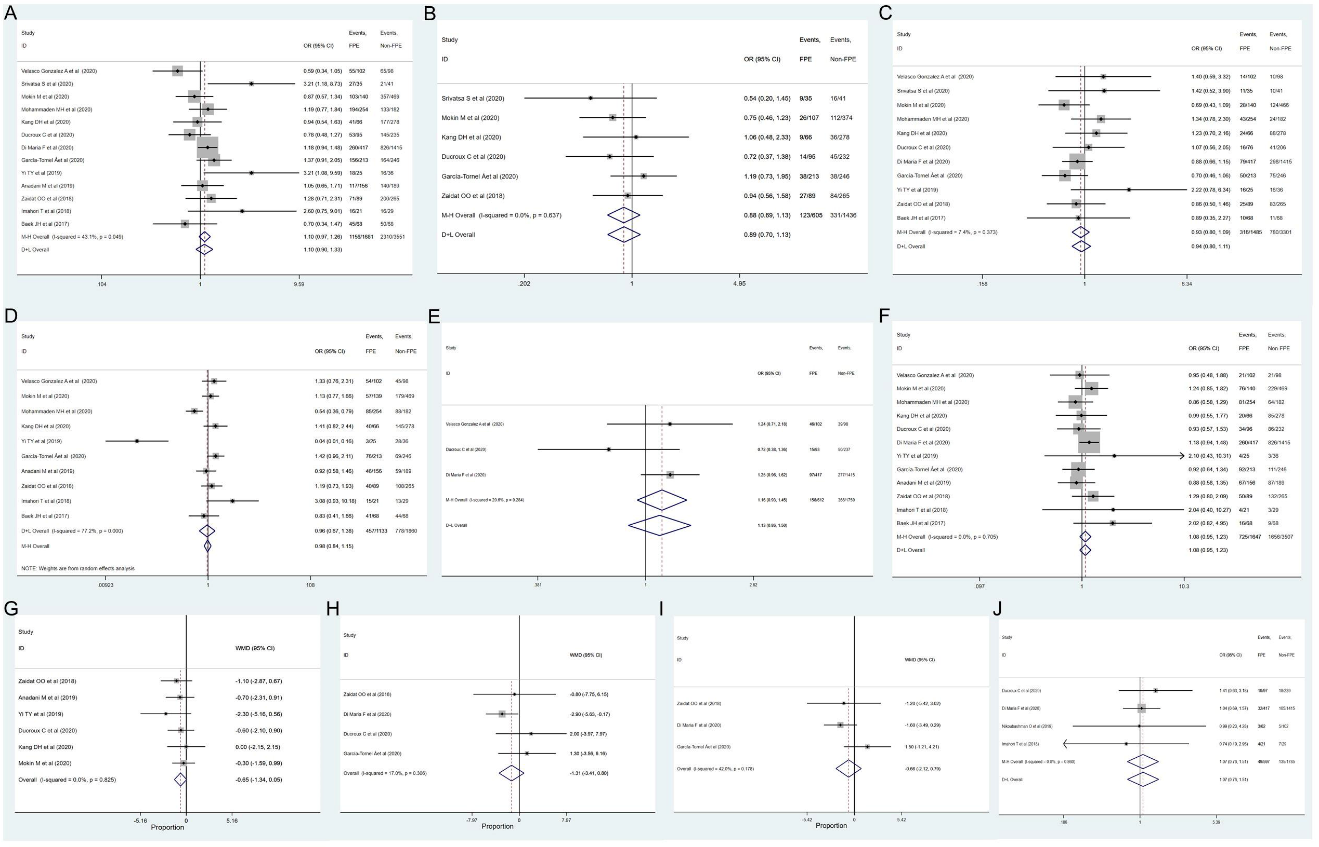


**Supplementary Fig. 1** The rest of influencing factors which were not significantly correlated with achieving successful recanalization with FPE. **a** hypertension; **b** CAD; **c** smoke; **d** atrial fibrillation; **e** previous anticoagulation therapy; **f** dyslipidemia; **g** initial NIHSS score; **h** systolic blood pressure; **i** diastolic blood pressure; **j** large artery atherosclerosis


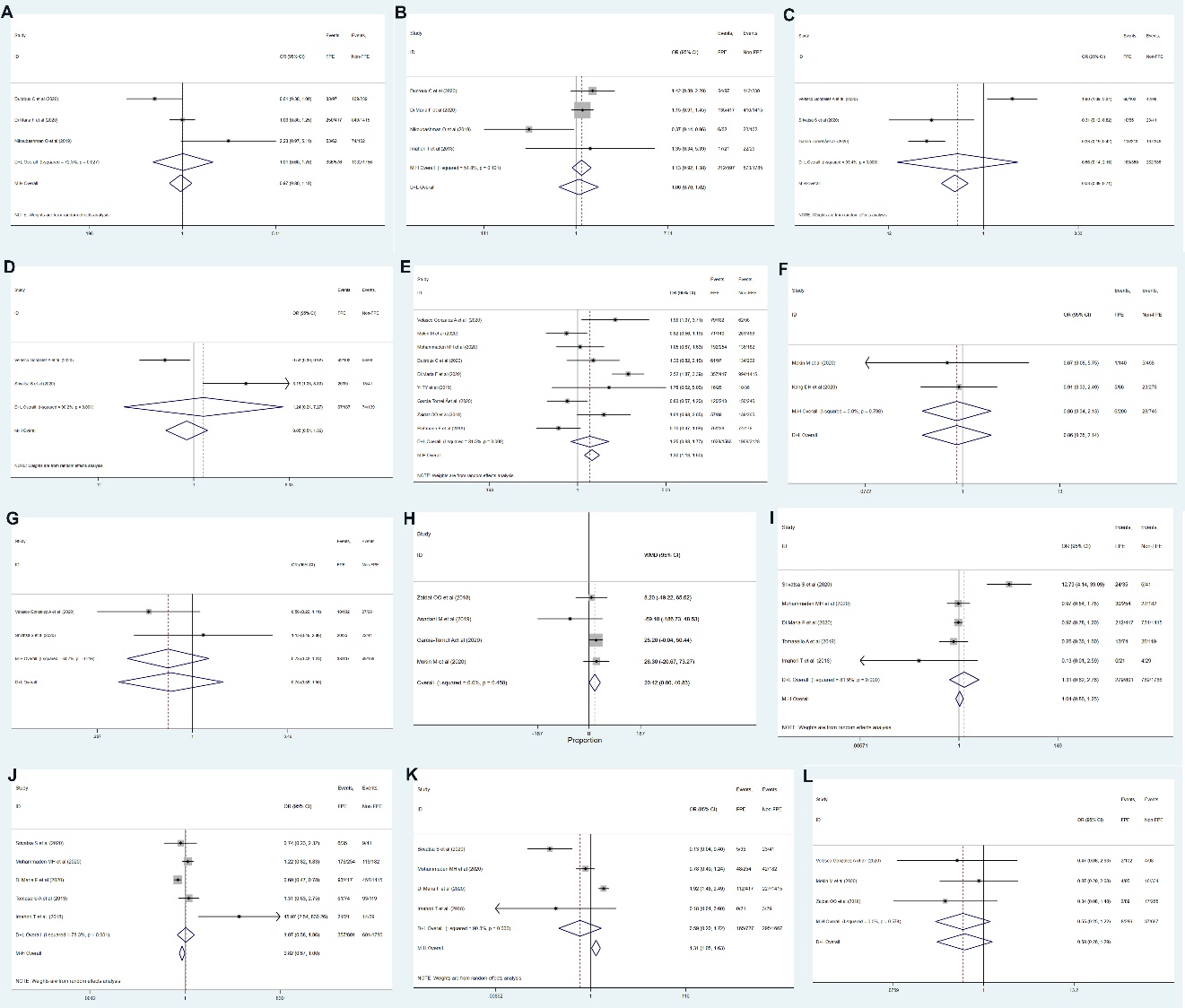


**Supplementary Fig. 2** The rest of influencing factors which were not significantly correlated with achieving successful recanalization with FPE. **a** cardioembolic etiology; **b** other etiology; **c** right side of occlusion; **d** left side of occlusion; **e** M1; **f** tandem occlusion; **g** ipsilateral AComA and PcomA; h time of onset to puncture; **i** aspiration only; **j** stent retriever only; **k** aspiration and stent retriever both**; l** migration to new territory.


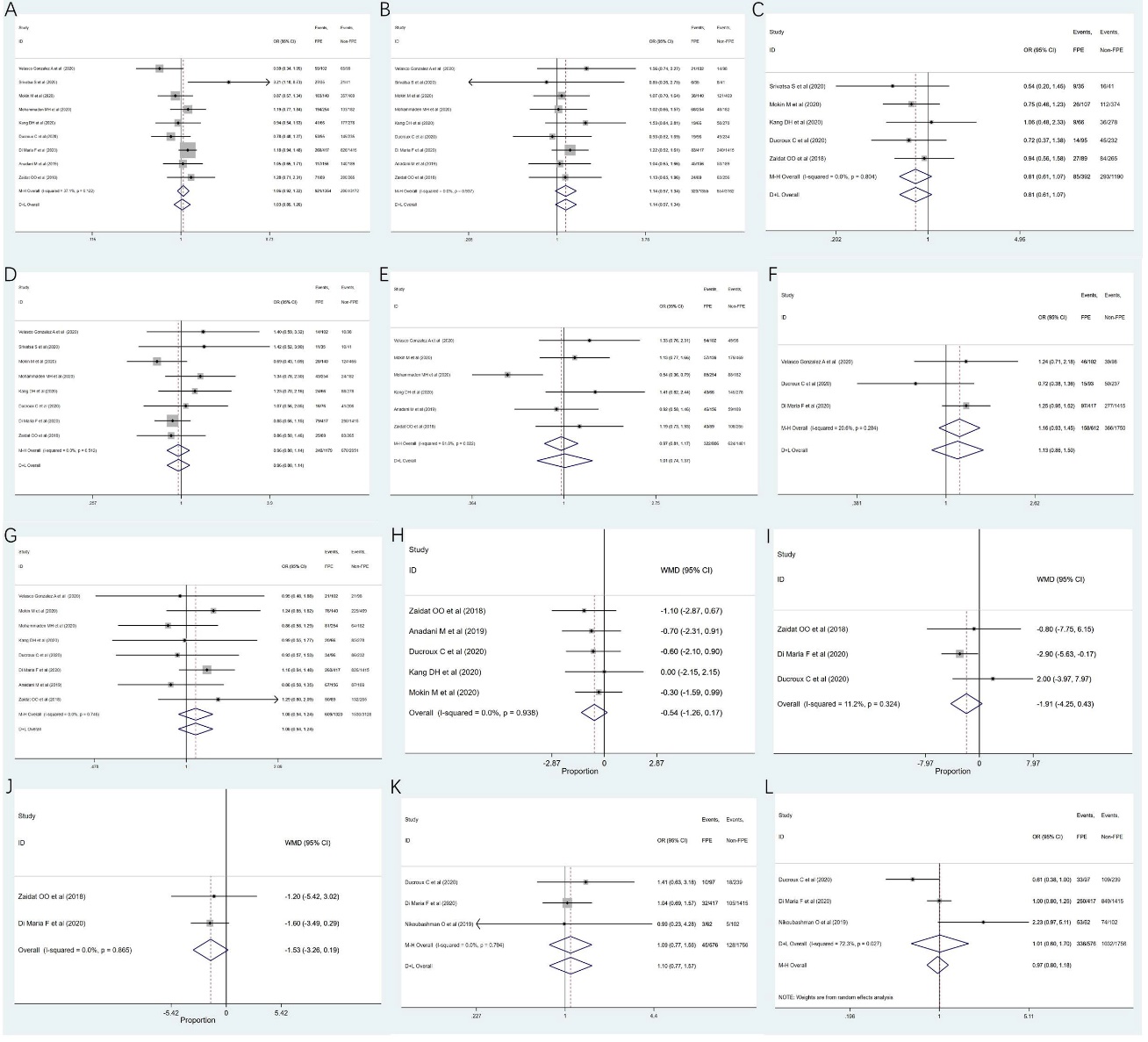


**Supplementary Fig. 3** The rest of influencing factors which were not significantly correlated with achieving complete recanalization with FPE. **a** hypertension; **b** diabetes mellitus; **c** CAD; **d** smoke; **e** atrial fibrillation; **f** previous anticoagulation therapy; **g** dyslipidemia; **h** initial NIHSS score; **i** systolic blood pressure; **j** diastolic blood pressure; **k** large artery atherosclerosis; **l** cardioembolic etiology


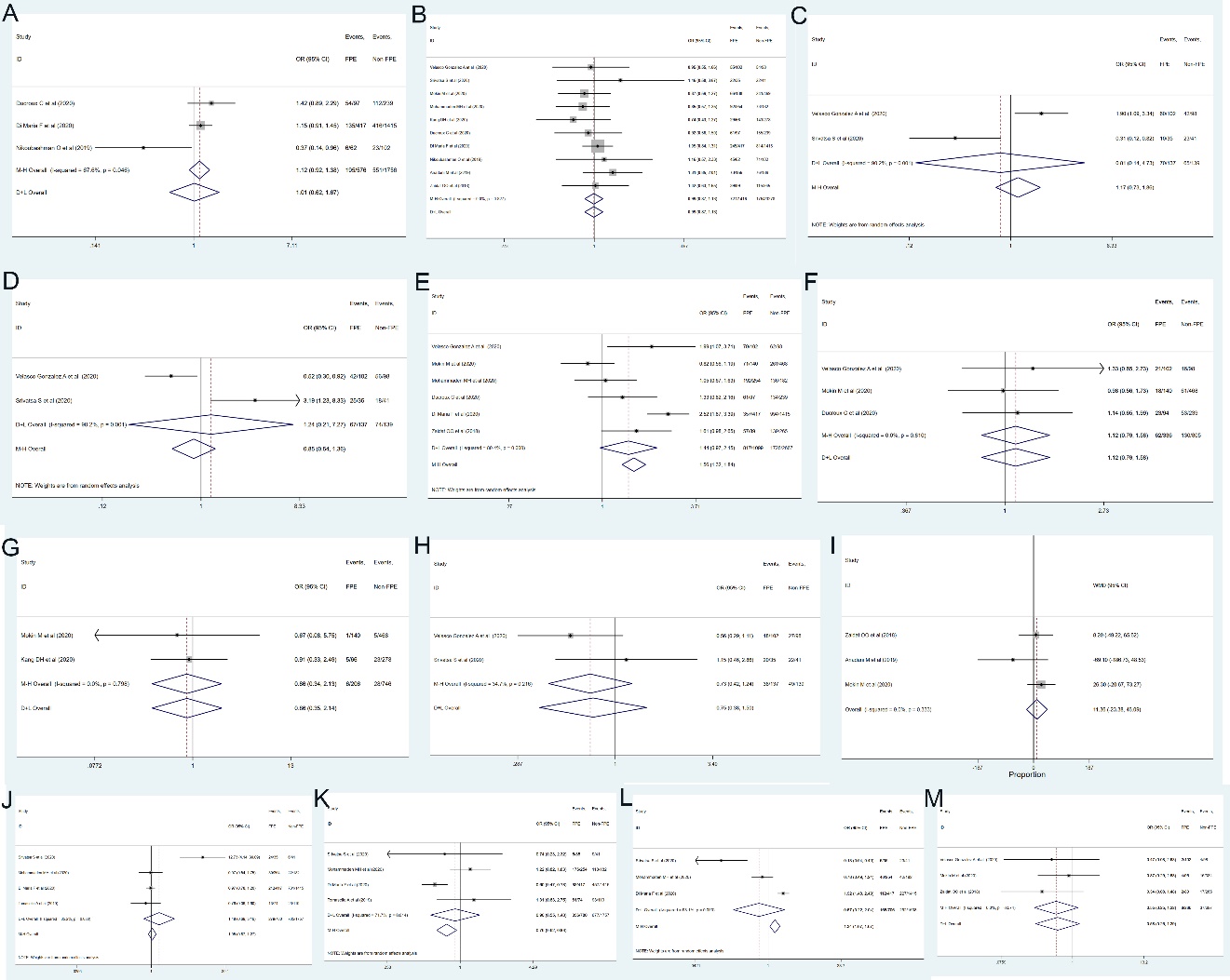


**Supplementary Fig. 4** The rest of influencing factors which were not significantly correlated with achieving complete recanalization with FPE. **a** other etiology; **b** IV thrombolysis; **c** right side of occlusion; **d** left side of occlusion; **e** M1; **f** M2; **g** tandem occlusion; **h** ipsilateral AComA and PcomA; **i** time of onset to puncture; **j** aspiration only; **k** stent retriever only; **l** aspiration and stent retriever both**; m** migration to new territory.


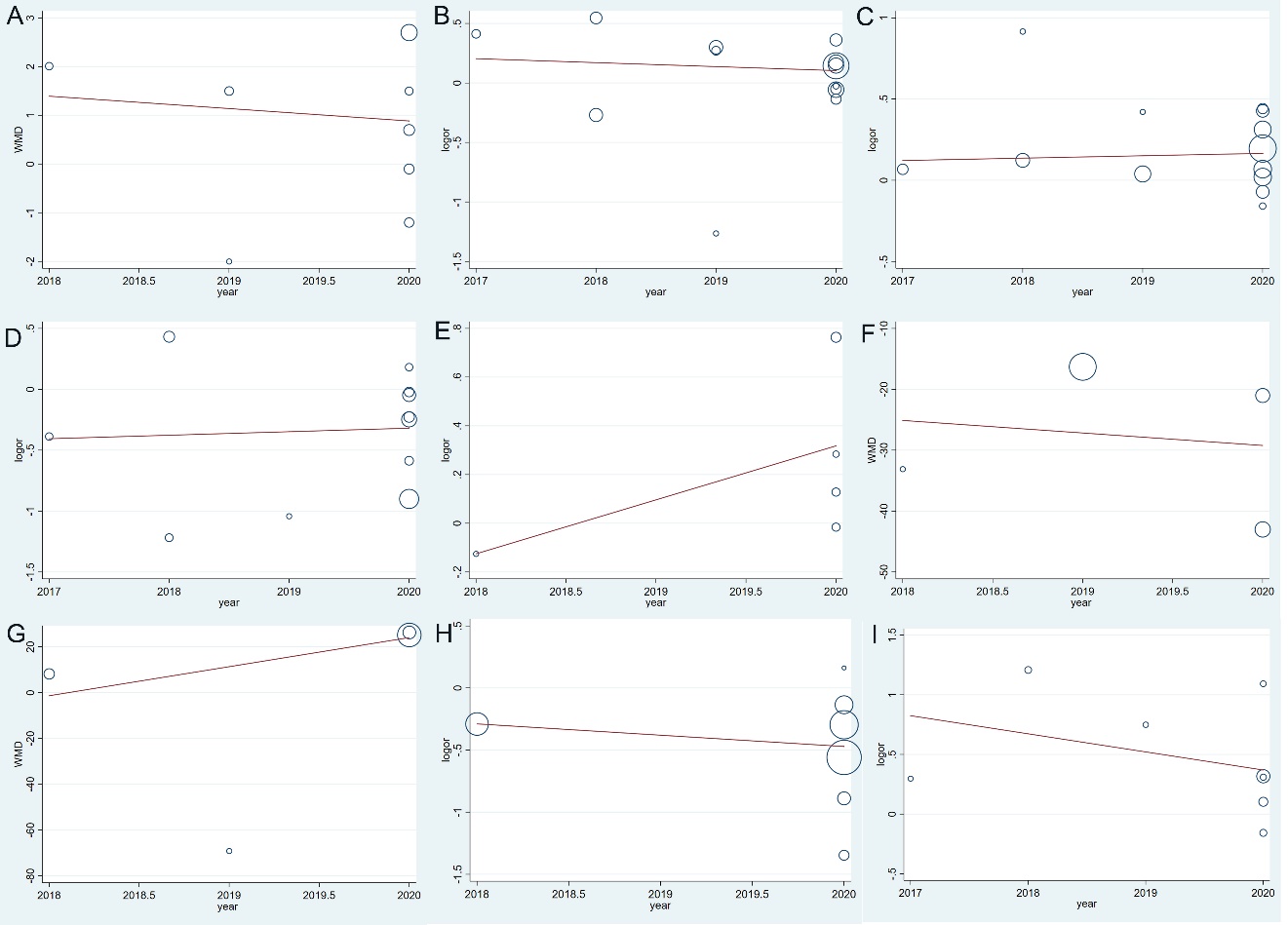


**Supplementary Fig. 5** Meta-regression analyses of outcomes of determinants for achieving successful recanalization with FPE **a** age; **b** female; **c** diabetes mellitus; **d** ICA; **e** M2; **f** duration of intervention; **g** time of onset to recanalization; **h** general anesthesia; **i** use of BGC.


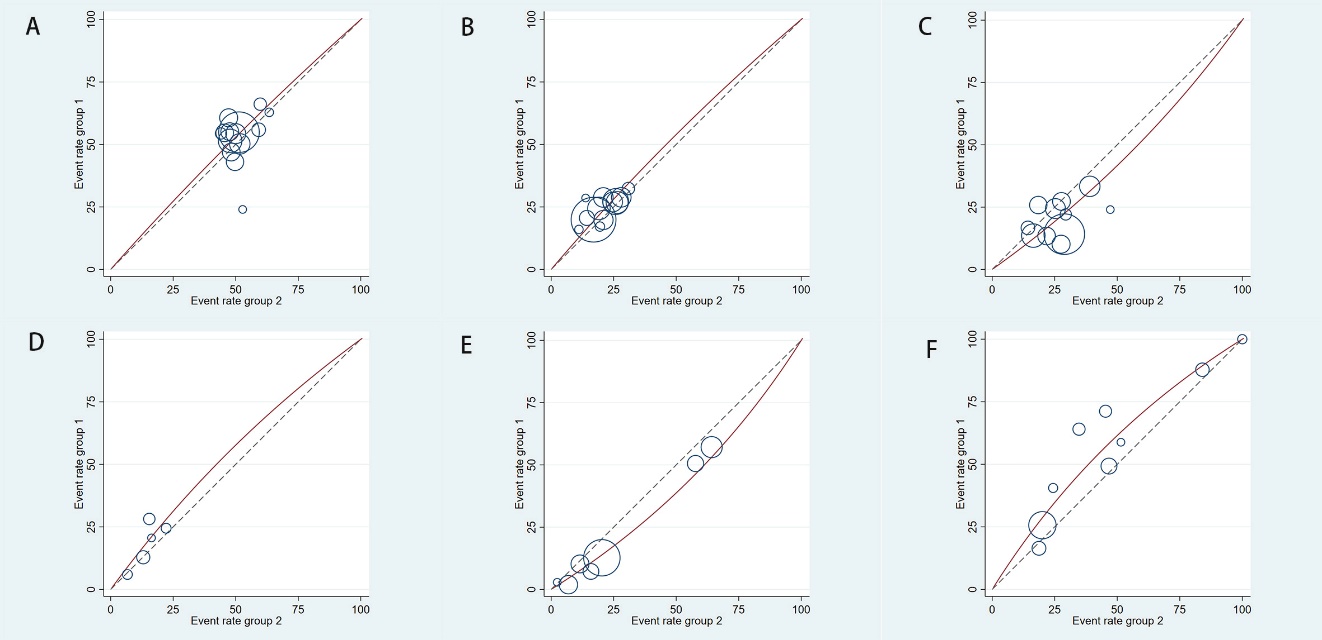


**Supplementary Fig. 6** Labbe plots of outcomes of determinants for achieving successful recanalization with FPE **a** female; **b** diabetes mellitus; **c** ICA; **d** M2; **e** general anesthesia; **f** use of BGC.


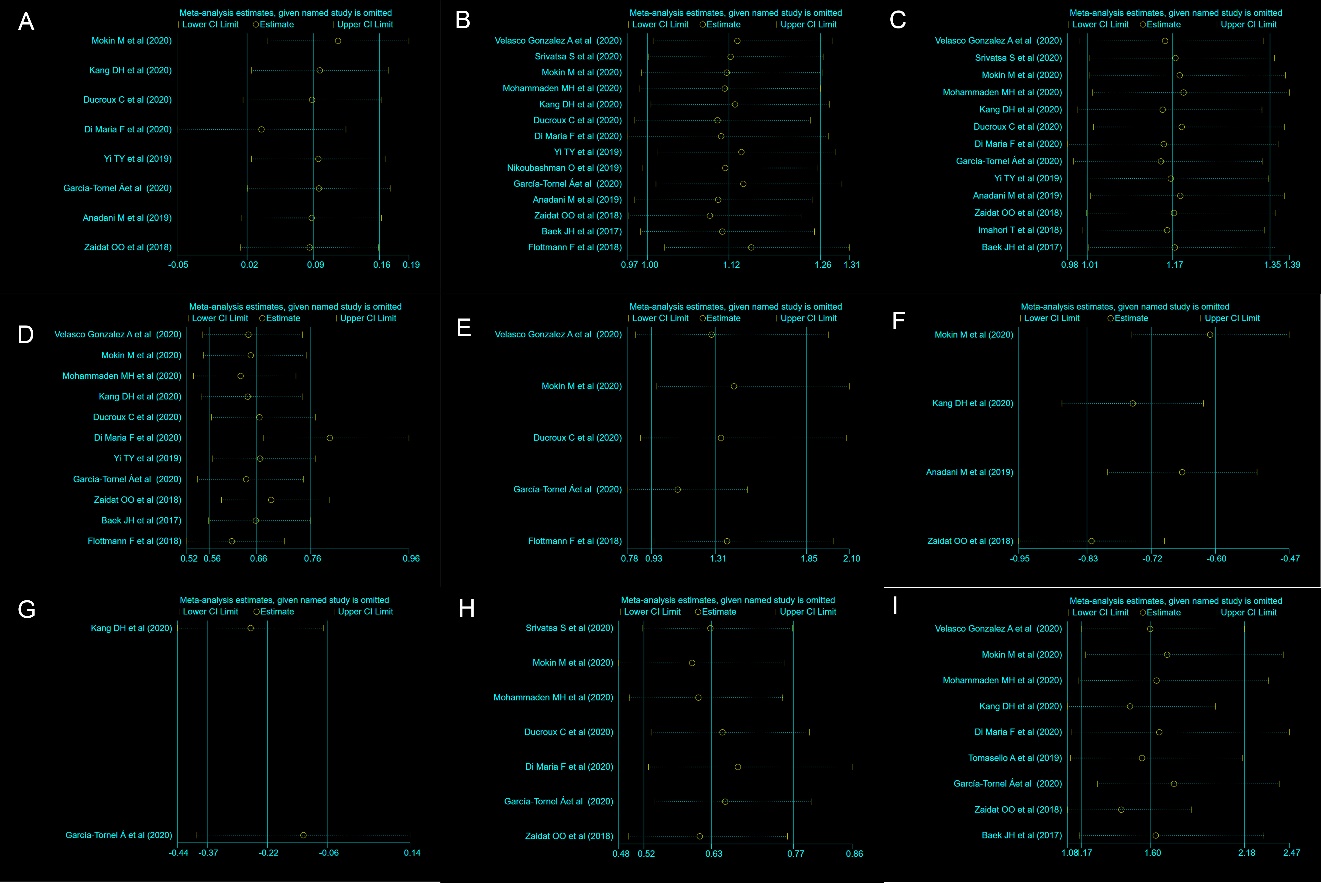


**Supplementary Fig. 7** Sensitivity analyses of outcomes of determinants for achieving successful recanalization with FPE **a** age; **b** female; **c** diabetes mellitus; **d** ICA; **e** M2; **f** duration of intervention; **g** time of onset to recanalization; **h** general anesthesia; **i** use of BGC.


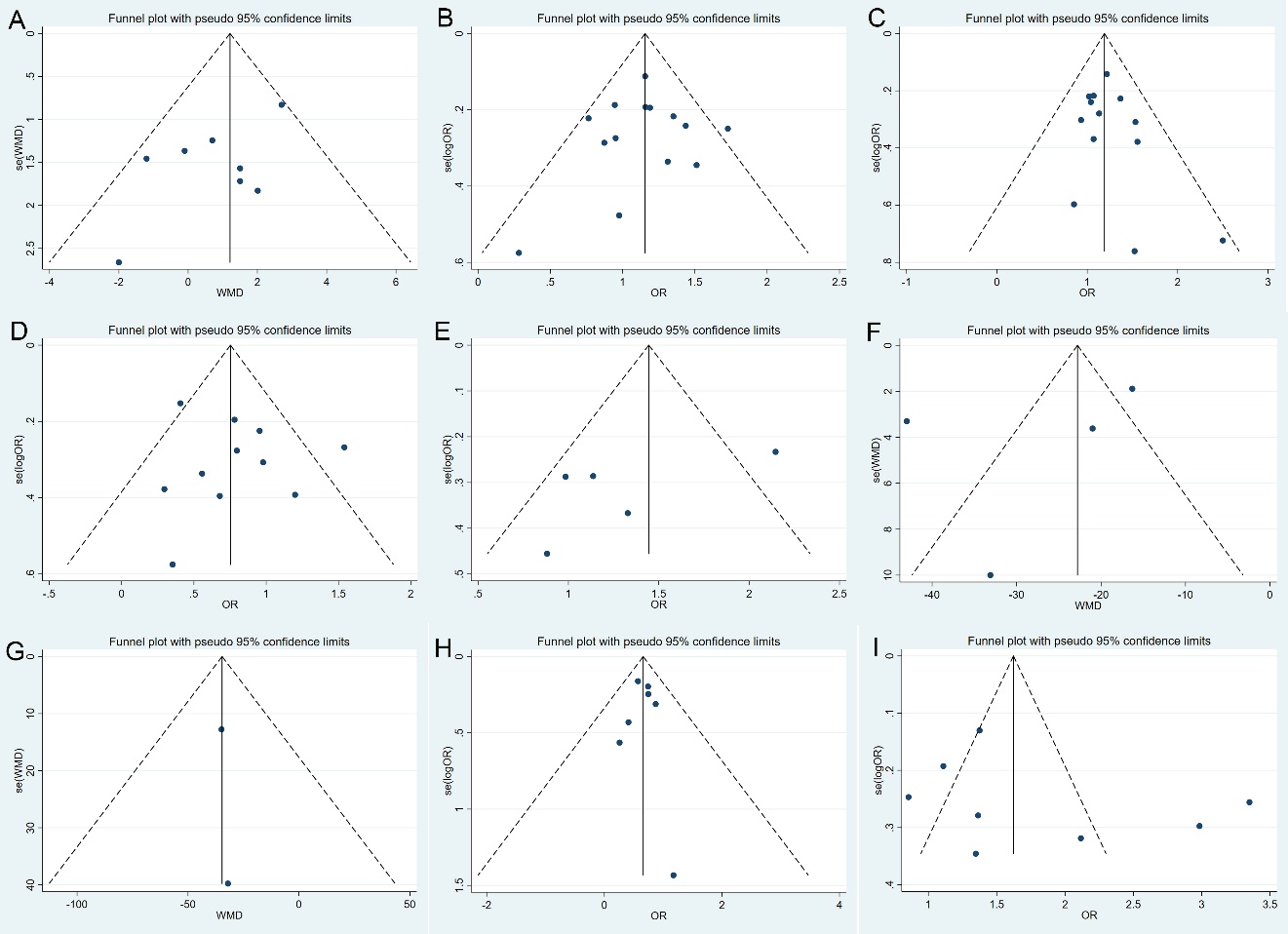


**Supplementary Fig. 8** Funnel plots of outcomes of determinants for achieving successful recanalization with FPE **a** age; **b** female; **c** diabetes mellitus; **d** ICA; **e** M2; **f** duration of intervention; **g** time of onset to recanalization; **h** general anesthesia; **i** use of BGC.


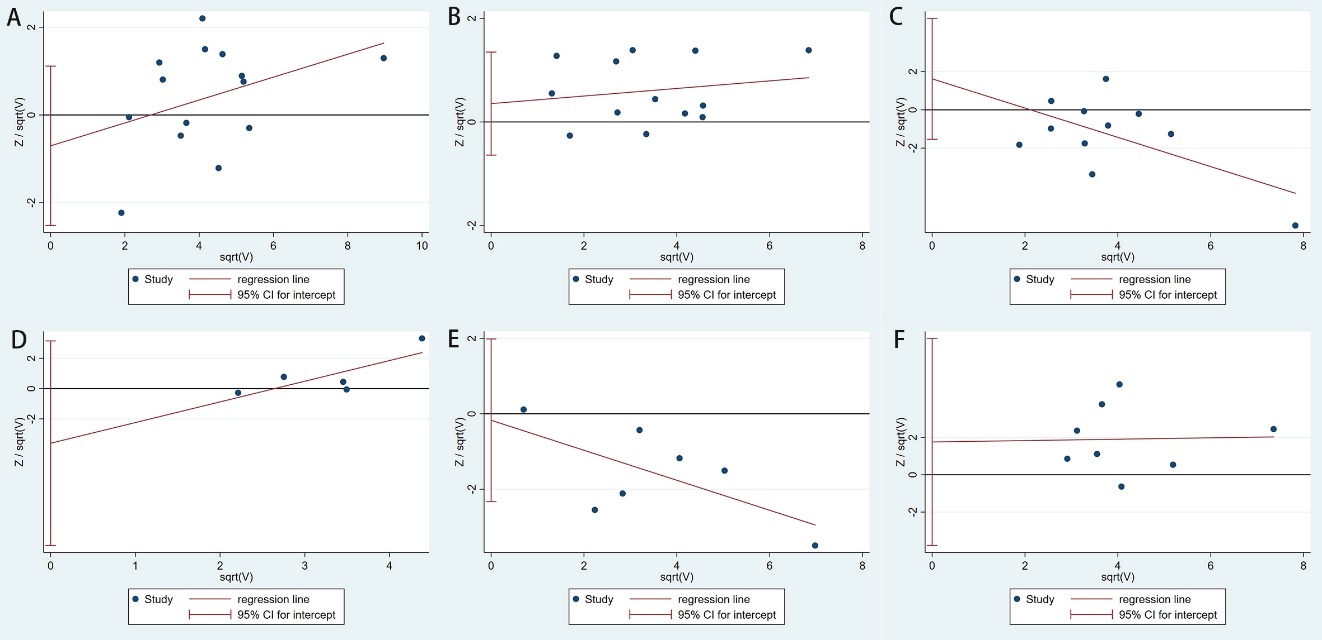


**Supplementary Fig. 9** Harbord funnel plots of outcomes of determinants for achieving successful recanalization with FPE **a** female; **b** diabetes mellitus; **c** ICA; **d** M2; **e** general anesthesia; **f** use of BGC.


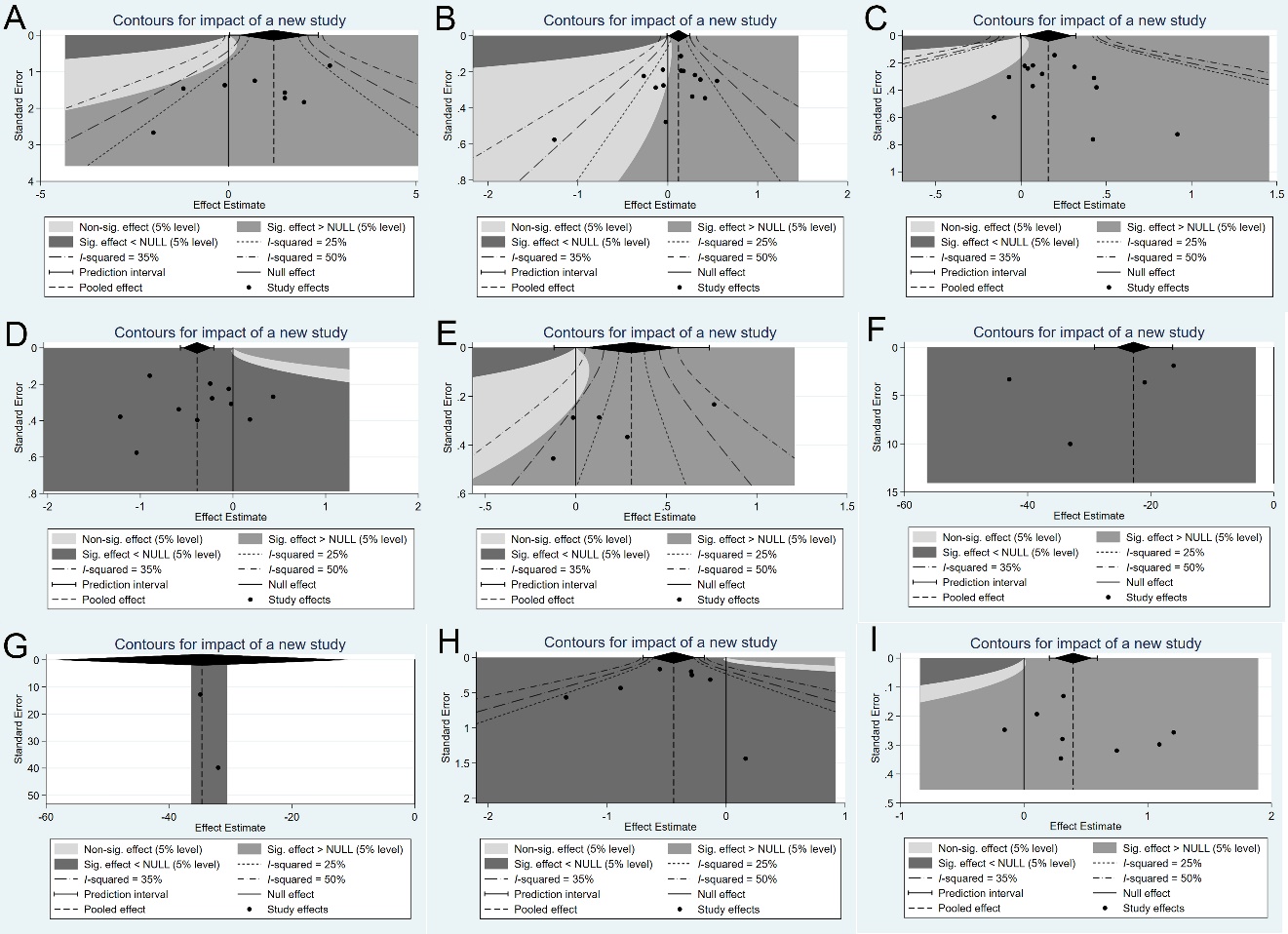


**Supplementary Fig. 10** Extfunnel plots of outcomes of determinants for achieving successful recanalization with FPE **a** age; **b** female; **c** diabetes mellitus; **d** ICA; **e** M2; **f** duration of intervention; **g** time of onset to recanalization; **h** general anesthesia; **i** use of BGC.


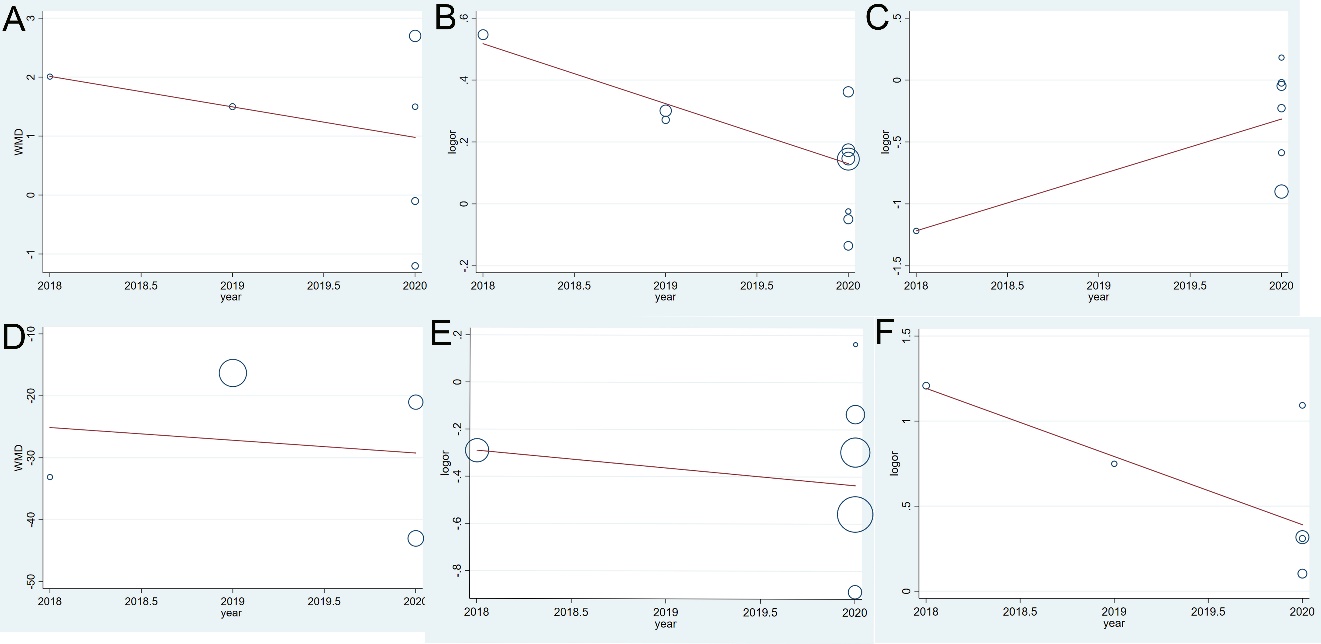


**Supplementary Fig. 11** Meta-regression analyses of outcomes of determinants for achieving complete recanalization with FPE **a** age; **b** female; **c** ICA; **d** duration of intervention; **e** general anesthesia; **f** use of BGC.


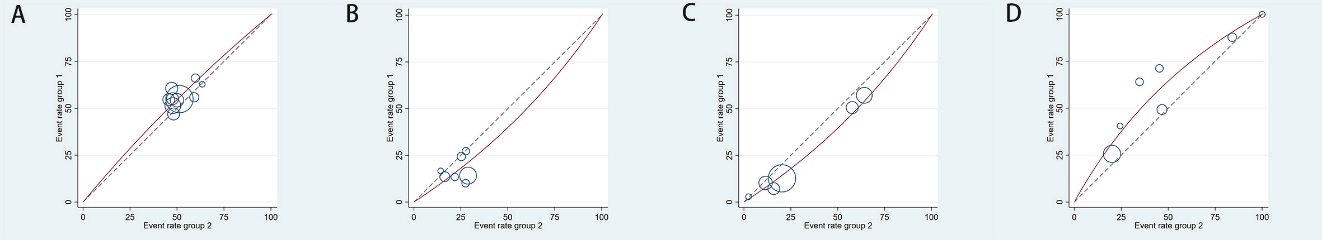


**Supplementary Fig. 12** Labbe plots of outcomes of determinants for achieving complete recanalization with FPE **a** female; **b** ICA; **c** general anesthesia; **d** use of BGC.


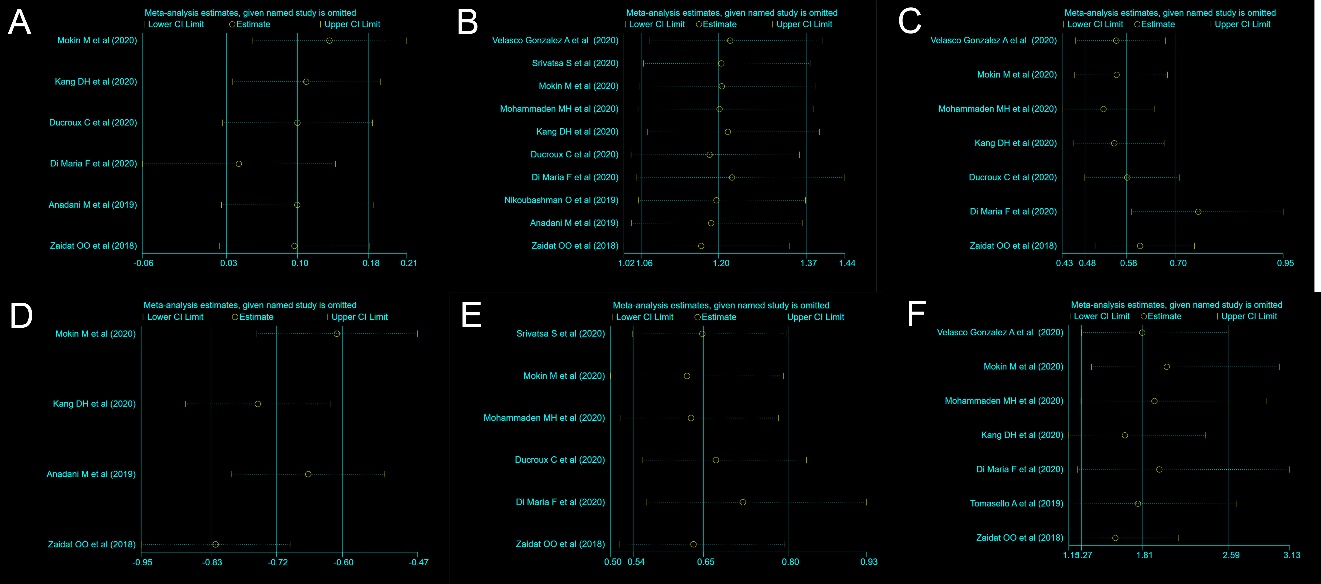


**Supplementary Fig. 13** Sensitivity analyses of outcomes of determinants for achieving complete recanalization with FPE **a** age; **b** female; **c** ICA; **d** duration of intervention; **e** general anesthesia; **f** use of BGC.


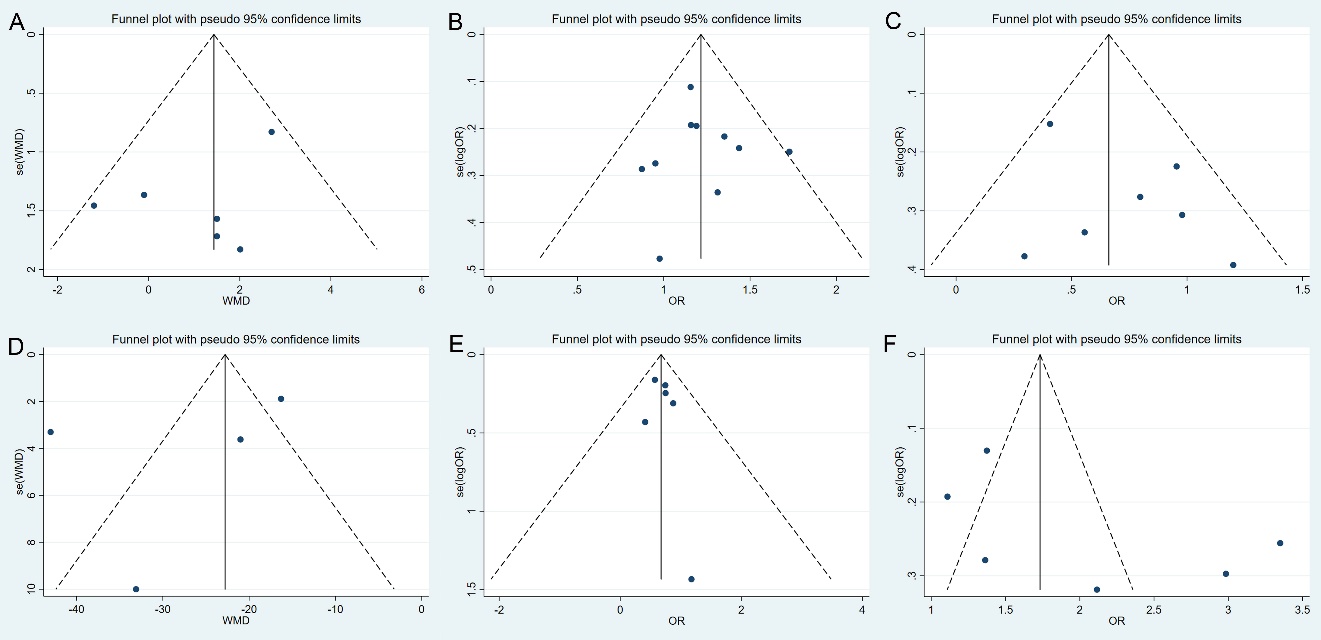


**Supplementary Fig. 14** Funnel plots of outcomes of determinants for achieving complete recanalization with FPE **a** age; **b** female; **c** ICA; **d** duration of intervention; **e** general anesthesia; **f** use of BGC.


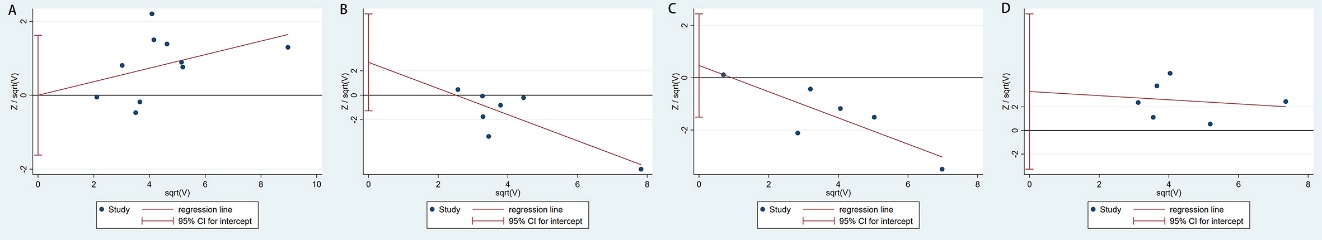


**Supplementary Fig. 15** Harbord funnel plots of outcomes of determinants for achieving complete recanalization with FPE **a** female; **b** ICA; **c** general anesthesia; **d** use of BGC.


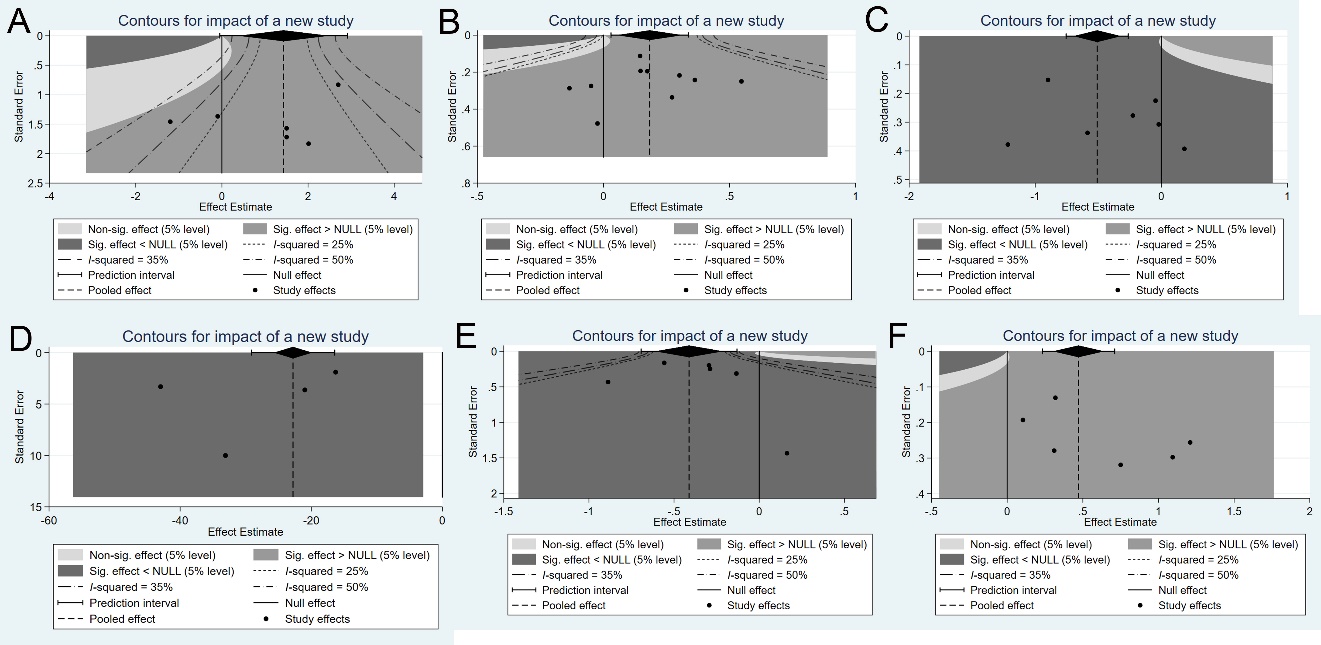


**Supplementary Fig. 16** Extfunnel plots of outcomes of determinants for achieving complete recanalization with FPE **a** age; **b** female; **c** ICA; **d** duration of intervention; **e** general anesthesia; **f** use of BGC.
